# Comparative Efficacy of Chinese Herbal Injections for Treating Acute Exacerbation of Chronic Obstructive Pulmonary Disease: A Bayesian Network Meta-Analysis of Randomized Controlled Trials

**DOI:** 10.1155/2018/7942936

**Published:** 2018-07-17

**Authors:** Xiaojiao Duan, Jiarui Wu, Xingyue Huang, Kaihuan Wang, Yi Zhao, Dan Zhang, Xinkui Liu, Xiaomeng Zhang

**Affiliations:** Department of Clinical Chinese Pharmacy, School of Chinese Materia Medica, Beijing University of Chinese Medicine, Beijing 100102, China

## Abstract

**Introduction:**

Acute exacerbation of chronic obstructive pulmonary disease (AECOPD) imposes a huge economic burden on healthcare systems worldwide. Chinese herbal injections (CHIs) are widely used to treat AECOPD. In this study, we examined the efficacy of CHIs in the treatment of AECOPD using a network meta-analysis (NMA).

**Methods:**

Literature search was conducted from electronic databases of randomized controlled trials (RCTs) on CHIs plus Western medicine (WM) versus WM. WinBUGS 1.4.3 and STATA 12.0 were adopted to compute calculations and prepare graphs, respectively.

**Results:**

We included 155 RCTs with 13,218 patients. The results revealed that Danhong injection (DH) + WM had the greatest therapeutic potential in terms of rate of clinical efficacy (RCE). In addition, in comprehensively improving RCE and FEV_1_%, and RCE and C-reactive protein, Huangqi injection (HQ) +WM was associated with preferable effects. Similarly, Xixinnao injection + WM, Reduning injection (RDN) +WM, and HQ+WM had a favorable effect on RCE and PaO_2_. The effect of RDN+WM was favorable in all outcomes except RCE. The safety of CHIs needs to be further assessed.

**Conclusions:**

Based on this NMA, DH+WM, HQ+WM, and RDN+WM were potential optimal therapies in AECOPD and their safety should be strictly monitored.

## 1. Introduction

Chronic obstructive pulmonary disease (COPD) is a lung disease characterized by progressive and persistent airflow limitation and abnormal inflammatory response in the airways [1, 2]. The estimated morbidity of COPD is up to 6.2% in nine Asia-Pacific territories and is a rapidly growing problem in Asia [3, 4]. Patients with worsening of their respiratory symptom beyond normal day-to-day variations and which lead to a change in medication are described as having an acute exacerbation of COPD (AECOPD) [2]. Frequent exacerbations of COPD are associated with increased mortality and AECOPD imposes a huge economic burden on healthcare systems worldwide [5, 6]. Although an inhaled bronchodilator is the first choice for treating AECOPD, there is no current consensus on the optimal benefits of pharmacological or nonpharmacological management [7, 8]. Recent surveys reported that traditional Chinese medicine is effective in relieving clinical symptoms, improving lung function, reducing inflammation, shortening acute exacerbation, and improving quality of life [9–12]. Moreover, it has an excellent safety profile [11]. Chinese herbal injection (CHI) is an innovative formulation with high bioavailability and rapid action [13]. Moreover, traditional pair-wise meta-analysis reviews manifested that numerous CHIs could be used to treat AECOPD with favorable efficacy [14–18]. Although their efficacy was confirmed, the optimal injection remains unclear because the traditional pair-wise meta-analysis can merely analyze the data from direct evidence and there was little evidence of direct comparison between two injections. Network meta-analysis (NMA) can achieve a comprehensive analysis through both direct and indirect comparisons and obtain the effects of interventions based on the probability of optimal treatment [19–21]. Therefore, to confirm the best therapy we must put the NMA into practice. In this study, we conducted a NMA to reveal which is the best CHI for treating AECOPD to provide evidence of evidence-based medicine for decision-making.

## 2. Methods

This NMA was conducted in accordance with the PRISMA Extension Statement for Reporting of Systematic Reviews Incorporating Network Meta-analyses of Health Care Interventions (Table S1) [22].

### 2.1. Eligibility Criteria and Exclusion Criteria

The eligibility criteria of this NMA conformed to the PICOS checklist [23].* Population.* Patients were diagnosed with AECOPD based on definite diagnostic standards.* Intervention and Control.* All patients were given conventional Western medicine (WM) treatment including inhaled bronchodilators (such as beta-agonists, antimuscarinic agents, and theophylline drugs), expectorants, and anti-inflammatory agents. The experimental group was treated with CHI combined with WM, and the control group received WM alone or WM plus another CHI.* Outcomes.* The primary outcome was the rate of clinical efficacy (RCE). RCE= (number of total patients - number of invalid patients)/number of total patients*∗*100%. Effectiveness status was classified as effective, valid, and invalid according to clinical symptoms and objective indicators. When clinical symptoms and objective indictors were unchanged or aggravated, patients were regarded as an invalid effectiveness status. Secondary outcomes were lung functions [ratio of forced expiratory volume in the first second to predicted value (FEV_1_%), ratio of forced expiratory volume in the first second to forced vital capacity (FEV_1_/FVC)], blood gas analysis indices [arterial partial pressure of carbon dioxide (PaCO_2_), arterial partial pressure of oxygen (PaO_2_), and blood pH], inflammatory markers [C-reaction protein (CRP), white blood cell count (WBCC), and neutrophil percentage (N%)], and adverse drug reactions/adverse drug events (ADRs/ADEs). Study design comprised randomized controlled trials (RCTs).

A RCT was excluded if it met any of the following criteria: (1) it was associated with any other diseases, such as AECOPD with heart failure or other pulmonary diseases; (2) effective evaluation criteria were not clear or consistent; (3) the course of treatment was not described; (4) there was no primary outcome; (5) it was repetitive; (6) the full text could not be obtained, or data were wrong and we received no response from the original author.

### 2.2. Search Strategy

We searched PubMed, the Cochrane Library, Embase, and several Chinese databases: China National Knowledge Infrastructure Database (CNKI), the Wan-Fang Database, the Chinese Scientific Journals Full-text Database, and the Chinese Biomedical Literature Database from initiation to Jan. 2nd, 2018. The references of the relevant literature were also checked. After the preanalysis, we included 12 different CHIs in the NMA: Chuankezhi injection (CKZ), Chuanxiongqin injection (CXQ), Danhong injection (DH), Huangqi injection (HQ), Reduning injection (RDN), Shenfu injection (SF), Shenmai injection (SM), Shengmai injection (SMI), Tanreqing injection (TRQ), Xuebijing injection (XBJ), Xixinnao injection (XXN), and Xiyanping injection (XYP). The specific search terms are shown in Table S2.

### 2.3. Data Extraction and Risk of Bias Assessment

All articles were managed by NoteExpress software (Wuhan University Library, Wuhan, China). Two researchers (XJD and XYH) screened potential studies according to the inclusion criteria by independently reading titles/abstracts and full text. Any discordance was resolved through group consensus or a third researcher (JRW). The data of enrolled studies were extracted in Microsoft Excel 2016 and included the first author, published year, baseline information (the number of patients, gender, age), details of interventions, outcomes and measured data, and factors of risk of bias.

Two authors (KHW and DZ) assessed the risk of bias in eligible studies independently by using the Cochrane Risk of Bias Tool [24]. Items evaluated were selection bias (random sequence generation and allocation concealment), performance bias (blinding of participants and personnel), detection bias (blinding of outcome assessment), attrition bias (incomplete outcome data), reporting bias (selective reporting), and other bias. There were three levels of bias, namely, “low risk”, “high risk”, and “unclear” for each item. Consensus was attained by discussion or getting a third opinion (JRW).

As the extracted data was acquired from published articles and private patient information was not obtained, so the ethical approval was not necessary.

### 2.4. Statistical Analysis

WinBUGS 1.4.3 software (MRC Biostatistics Unit, Cambridge, UK) and STATA 12.0 software (Stata Corporation, College Station, TX, USA) were adopted to compute calculations and prepare graphs, respectively. The odds ratios (ORs) were calculated to determine the effect for dichotomous outcomes. Secondary outcome measures were continuous variables, from which mean differences (MDs) were calculated. The 95% confidence intervals (CIs) were measured to estimate uncertainty. When 95% CIs of ORs did not cover one and 95% CIs of MDs did not contain zero, differences between the groups were considered statistically significant. The Markov chain Monte Carlo method with random-effect model was performed using the WinBUGS software to carry out the network meta-analysis. When running the WinBUGS, 10,000 iteration was set to estimate the pooled effect measure and the first 5,000 was used for burn-in to eliminate the impact of the initial value. Network diagrams of different outcomes were drawn by the STATA software to present relationships with the selected CHIs. The results of the WinBUGS software calculations were employed by the STATA software to calculate surface under the cumulative ranking probabilities (SUCRA). An intervention resulting in a larger SUCRA was considered to be the more effective treatment. Therefore, SUCRA was used to evaluate the ranking probabilities for each treatment. A comparison-adjusted funnel plot was used to identify publication bias. If the point distributed in the funnel was symmetrical, there was no publication bias [25]. The method of clustering analysis was utilized to comprehensively compare the effect of CHIs on two different outcomes [26].

## 3. Results and Discussion

### 3.1. Literature Retrieval and Study Characteristics

**Figure 1:**
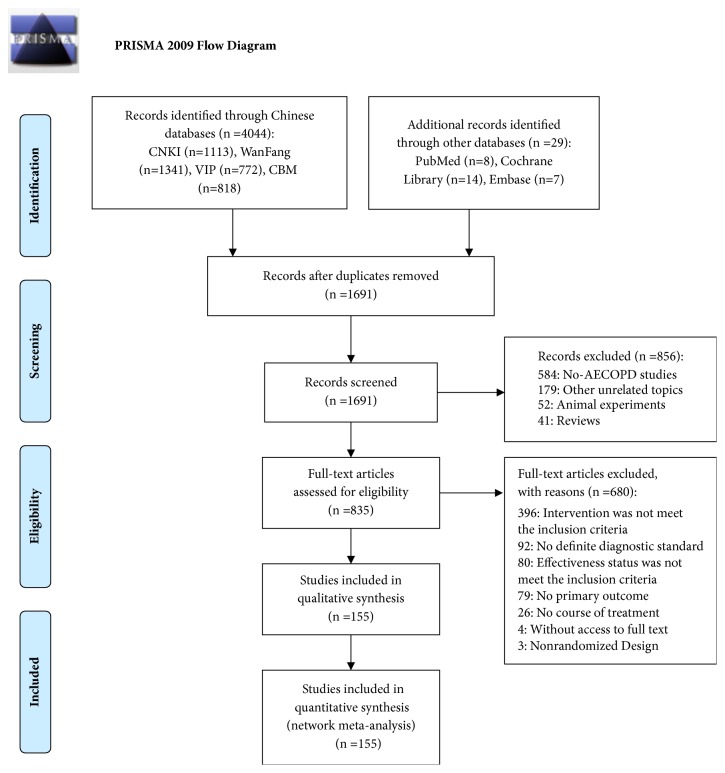
Flow diagram of study search (n, number of articles; CNKI, China National Knowledge Infrastructure Database; WanFang, the Wan-Fang Database; VIP, the Chinese Scientific Journals Full-Text Database; CBM, the Chinese Biomedical Literature Database).

**Figure 2:**
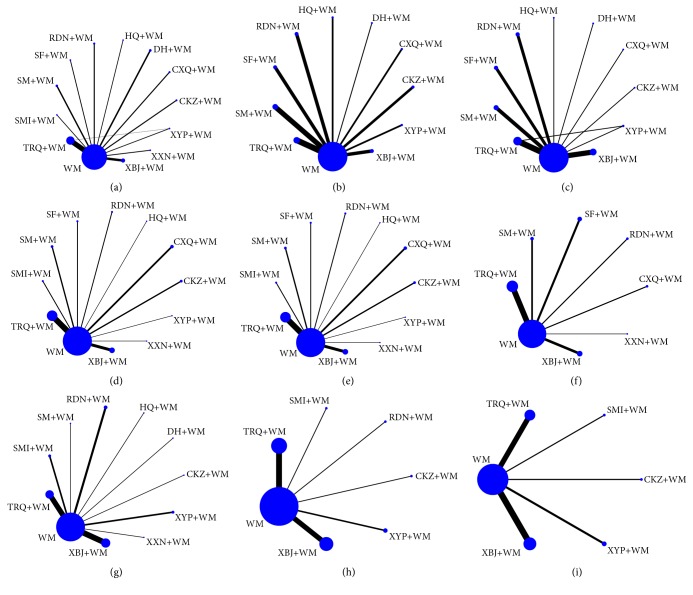
Network graph for different outcomes ((a) RCE; (b) FEV_1_%; (c) FEV_1_/FVC; (d) PaCO_2_; (e) PaO_2_; (f) pH; (g) CRP; (h) WBCC; (i) N%).

A total of 4,073 articles were identified from the aforementioned electronic databases. After duplications were removed, 856 studies were screened by titles/abstracts and 680 by full-texts. Eventually, 155 RCTs were remained with 154 indirect comparisons and 1 direct comparison (TRQ+WM versus XYP+WM). Further details of the literature screening process are presented in Figure 1. All the eligible studies were conducted in China from 2000 to 2017. Twelve CHIs were included in the analysis, namely, TRQ, XBJ, DH, SM, RDN, CXQ, CKZ, XYP, SF, XXN, HQ, and SMI. The network graphs of the 12 CHIs with different outcomes are depicted in Figure 2.

The 155 enrolled studies involved 13,218 patients, with 6,999 in the experimental group, and 6,519 in the control group. Besides two studies that did not report age composition, there were 8,252 male patients, accounting for 63.03% (8,252/13,093). The maximum sample size was 120 and the minimum was 15. All the studies reported RCE, and 33 (21.3%), 34 (21.9%), 40 (25.8%), 40 (25.8%), 28 (18.1%), 36 (23.2%), 20 (12.9%), and 15 (9.7%) RCTs reported the FEV_1_%, FEV_1_/FVC, PaCO_2_, PaO_2_, pH, CRP, WBCC, and N%, respectively. The details of study characteristics are depicted in Table S3.

### 3.2. Risk of Bias Assessment

**Figure 3:**
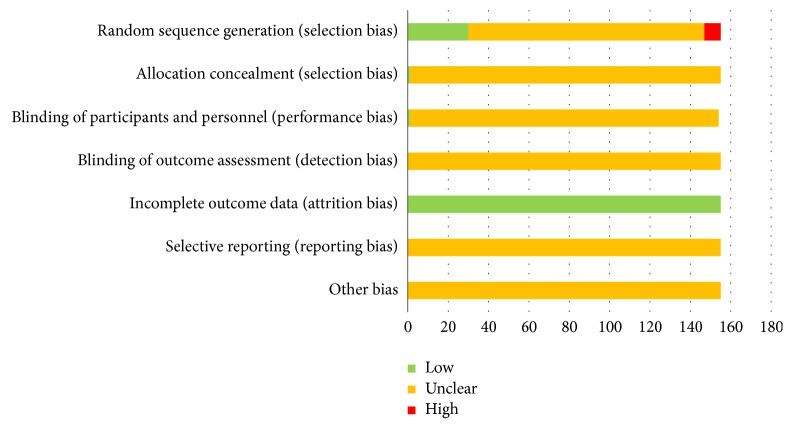
Assessment of risk bias.

In terms of bias assessment, 30 of the 155 studies adequately described their methods to generate the random sequence, so their selection bias was considered to be low risk due to adequate generation of a randomized sequence. Moreover, 8 studies were grouped in order of admission, and they were assessed as high risk. The remaining studies only described “random”, so they were deemed as unclear risk. In terms of allocation concealment, only 1 study ensured allocation concealment during the implementation process; thus it was estimated as low risk. The others were unclear risk. We rated 2 studies as low risk for performance bias, because they used a single blind method in their study approach. None of the studies had incomplete data, so the attrition bias was evaluated as low risk. Detection bias, report bias, and other bias were determined as unclear risk when too few details were available to make a decision either way. In summary, the quality of the eligible studies was not high (see Figure 3).

### 3.3. Results of the Network Meta-Analysis

#### 3.3.1. Results of Bayesian Network Meta-Analysis

A total of 155 RCTs involving 12 CHIs reported the RCE. The ORs with 95% CIs for each of the CHIs for RCE are presented in Table S4. In terms of RCE, TRQ+WM, XBJ+WM, DH+WM, SM+WM, RDN+WM, CXQ+WM, CKZ+WM, SF+WM, XXN+WM, XYP+WM, HQ+WM, and SMI+WM resulted in a significantly better outcome than WM alone. In addition, DH+WM was more efficacious than SF+WM. The differences between the above groups were statistically significant. No statistically significant difference was observed between the other interventions.

The lung functions that this NMA investigated were FEV_1_% and FEV_1_/FVC. This two outcomes involved 10 CHIs, except XXN and SMI. Of the 155 RCTs, 35 provided data on FEV_1_% and 34 provided data on FEV_1_/FVC. The FEV_1_% results indicated that SM+WM, RDN+WM, and HQ+WM were more effective than WM alone. Likewise, SM+WM yielded a better result than XYP+WM, and there was a significant difference between the two groups. No significant differences were found between the other groups for the remaining treatments. In terms of FEV_1_/FVC, TRQ+WM was the only therapy that was significantly better than WM and the difference was significant. The MDs and 95% CIs for the lung function tests are depicted in Table S5.

For blood gas indices, PaCO_2_, PaO_2_, and pH, were reported in 40, 40, and 28 RCTs, respectively. The RCTs that reported PaCO_2_ and PaO_2_ included 11 CHIs, except DH, and the RCTs that reported pH included 7 CHIs, namely, TRQ, XBJ, SM, RDN, CXQ, SF, and XXN. The results of the NMA for PaCO_2_ revealed that SMI+WM showed significant benefits for PaCO_2_ when compared with WM (MD=-14.40, 95% CI: -25.98, -2.96). There was no statistical difference between the other interventions. In the case of PaO_2_, patients who received TRQ+WM had better PaO_2_ than those treated with WM alone (MD=1.16, 95% CI: 5.60, 10.24), and the difference between the groups was statistically significant. No significant differences were found for the different treatments with respect to pH.

With respect to the inflammatory markers, CRP was reported in 36 RCTs with 10 included CHIs, WBCC was reported in 20 RCTs with 6 included CHIs, and N% was reported in 15 RCTs with 5 included CHIs. No significant difference in inflammatory markers was found between any of the treatments.

#### 3.3.2. Results of Rank Probability Based on SUCRA

The ranks for interventions based on SUCRA for each outcome are displayed in Table 1. All treatment options were better than WM in overall outcome.

For RCE, the rank of CHIs was DH+WM (85.5%, 13 RCTs), HQ+WM (76.7%, 6 RCTs), CXQ+WM (69.4%, 9 RCTs), XXN+WM (67.8%, 7 RCTs), SM+WM (59.5%, 12 RCTs), TRQ+WM (55.2%, 48 RCTs), RDN+WM (54.5%, 10R CTs), XBJ+WM (51.6%, 22 RCTs), SMI+WM (39.5%, 6 RCTs), XYP+WM (38.8%, 6 RCTs), CKZ+WM (32.4%, 9 RCTs), and SF+WM (19.1%, 7 RCTs).

The top four CHIs for FEV_1_% were SM+WM (76.6%, 6 RCTs), RDN+WM (75.1%, 4 RCTs), HQ+WM (69.0%, 2 RCTs), and XBJ+WM (58.8%, 4 RCTs). Similarly, with FEV_1_/FVC, RDN+WM (73.6%, 4 RCTs) was ranked the highest, followed by XBJ+WM (67.6%, 7 RCTs), SM+WM (66.9%, 5 RCTs), and TRQ+WM (58.3%, 9 RCTs).

SMI+WM (83.6%, 2 RCTs) showed the most favorable response for PaCO_2_ followed by RDN+WM (75.2%, 2 RCTs), HQ+WM (59.1%, 1 RCT), and SM+WM (54.9%, 3 RCTs). Furthermore, XXN+WM (73.8%, 1 RCT) was shown to be the best intervention to improve PaO_2,_ followed by RDN+WM (71.9%, 2 RCTs), HQ+WM (60.9%, 1 RCT), and SMI+WM (56.1%, 2 RCTs). As for pH, the top three CHIs were ranked as follows: RDN+WM (70%, 2 RCTs), XXN+WM (61%, 1 RCT), and SM+WM (58.4%, 3 RCTs).

The CHIs were also ranked based on the SUCRA for CRP and the top four therapies were TRQ+WM (63.3%, 10 RCTs), HQ+WM (60.6%, 1 RCT), XXN+WM (59.2%, 1 RCTs), and SMI+WM (57.4%, 3 RCTs). In terms of WBCC, RDN+WM (67%, 1 RCT), XBJ+WM (59.6%, 7 RCTs), and SMI+WM (58%, 1 RCT) achieved the most positive effect and CKZ+WM (84.1%, 1 RCT), SMI+WM (66.1%, 1 RCT), and XBJ+WM (56.5%, 6 RCTs) had the best outcome for N%.

**Figure 4:**
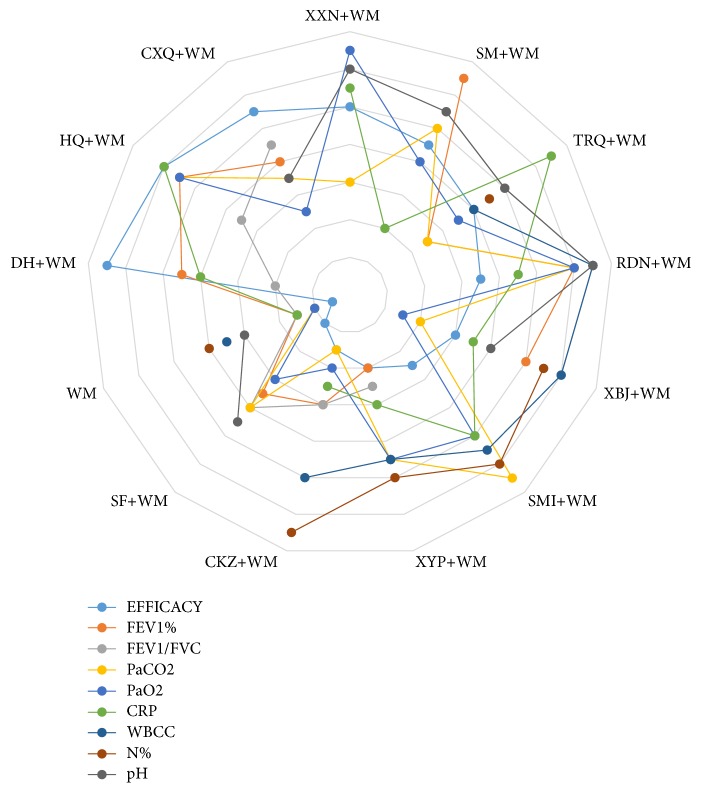
Radar map of ranking of treatment options relative to nine outcomes based on SUCRA. (If the intervention resulted in a favorable outcome, then the point is close to the outside of the map.)

Figure 4 showed the ranking of the 13 treatment measures for different outcomes. This figure showed that the rank of RDN+WM was better with most outcomes, and it would seem a likely candidate for optimal treatment of AECOPD.

### 3.4. Cluster Analysis

**Figure 5:**
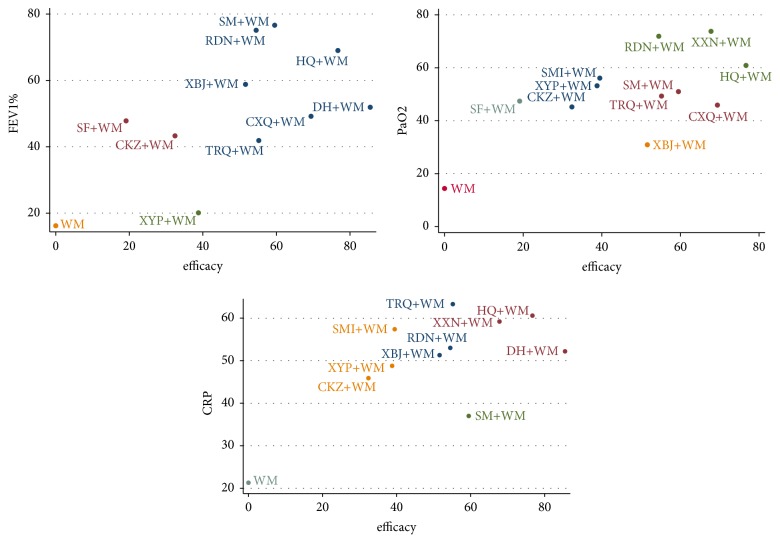
Cluster analysis plot for four outcomes. (Interventions with the same colour belonged to the same cluster, and interventions located in the upper right corner indicate optimal therapy for two different outcomes.)

Cluster analysis was performed on RCE and FEV_1_% for lung function, RCE and PaO_2_ for blood gas analysis indices, and RCE and CRP for inflammatory markers, so as to comprehensively compare the efficacy of CHIs in two different outcomes.  Figure 5 shows that HQ+WM had the most favorable response for RCE and FEV_1_%; XXN+WM, HQ+WM, and RDN+WM achieved the best outcomes with respect to RCE and PaO_2_; and HQ+WM had the most favorable influence with respect to RCE and CRP.

### 3.5. Publication Bias

**Figure 6:**
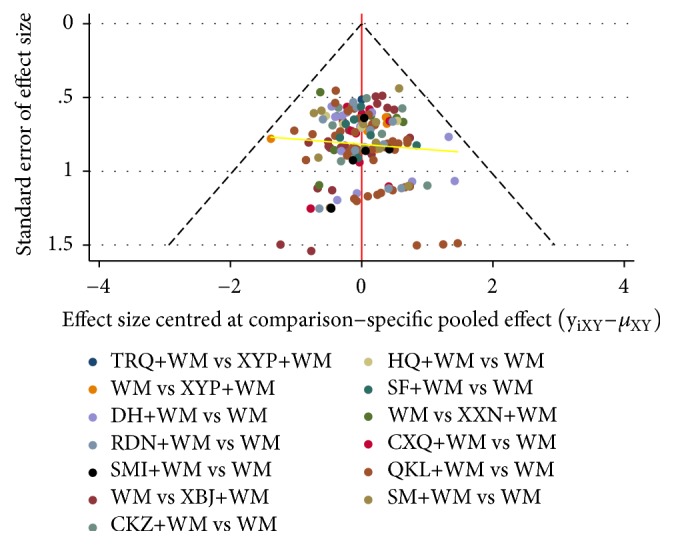
Comparison-adjusted funnel plot for the rate of clinical efficacy. (Points with different colours represent different interventions. If the points distributed in the funnel are symmetrical, there is no publication bias.)

According to the primary outcome, STATA software was used to draw a comparison-adjusted funnel plot so as to evaluate publication bias. As shown in Figure 6, the location of points in the funnel plot was basically symmetrical based on the midline, and the adjusted auxiliary line was almost perpendicular to the midline, suggesting the publication bias of this study was small.

### 3.6. Safety

Among 155 included RCTs, 91 RCTs did not address ADRs or ADEs, 38 studies clearly indicated that there were no significant ADRs/ADEs, and the ADRs/ADEs occurring during the implementation of trials were reported in the residual 26 studies. The details of ADRs/ADEs are represented in Table 2. Because the majority of eligible studies did not focus on the monitoring of ADRs/ADEs, so the safety of these treatments needs to be further explored.

### 3.7. Discussion

Based on the data from the 155 enrolled RCTs, the results of this NMA revealed that DH+WM showed the greatest treatment potential with respect to RCE. In addition, HQ+WM was found to be associated with a comprehensive improvement of RCE and FEV_1_% and RCE and CRP. Similarly, XXN+WM, RDN+WM, and HQ+WM were associated with a positive effect on RCE and PaO_2_. RDN+WM positively affected all outcomes except RCE and is worthy of attention. Different injections should be selected for different therapeutic purposes. With respect to safety, 58.71% (91/155) of the trials did not report ADRs/ADEs and indicated a need for improvement of monitoring of ADRs/ADEs in the treatment. Of the 64 studies that did report on ADRs/ADEs, only 40.63% (26/64) of the RCTs adequately described the ADRs/ADEs during the treatment, highlighting the need for more attention to be given to the safety of therapies used for AECOPD. Most of the ADRs/ADEs were in line with their specification, but flushing with DH and vasculitis with XBJ are unknown side effects of them. The flushing observed with DH has been reported in other literature [27], whereas vasculitis has not been reported in association with XBJ. The safety of XBJ needs to be further explored. We displayed the common ADRs of 12 CHIs based on specification and literature search in Table S6. It showed that pruritus, skin rash, chest congestion, and gastrointestinal adverse reactions were the most common ADRs. According to the drug dosage instructions, 1 RCT on TRQ, 2 RCTs on XBJ, 3 RCTs on RDN, 7 RCTs on CXQ, 4 RCTs on HQ, and 1 RCT on SMI gave an overdose of the treatment. In these 18 studies, 11 (61.11%) did not report ADRs/ADEs, and monitoring of ADRs/ADEs was not stringent when this ‘super' dosage was used. Given this, we suggest that ADRs/ADEs should be monitored at all times during treatments, especially in the first 30 minutes. In addition, the ADRs/ADEs described in the side effects listed for the medicine should be strictly monitored.

In this study, the efficacy and safety of 12 CHIs for treating AECOPD were evaluated in 155 RCTs by NMA, and 9 different outcomes were included. The 12 CHIs were selected from all the CHIs that conform to national drug standards in China and based on the number of eligible RCTs. As the vast majority of RCTs relating to CHIs were published in Chinese journals, the initial retrieval was conducted in the CNKI database only. A total of 132 CHIs and 36 chemical injections where the main ingredients were extracted from traditional Chinese medicine were searched in CNKI, and if the relevant RCTs were less than five, the CHI was removed. The 12 selected CHIs can be divided into three categories from the perspective of traditional Chinese medicine: TRQ, RDN, and XYP were placed in the category of clearing heat; DH, CXQ, and XBJ were in the category of promoting blood circulation; and SM, SMI, HQ, and SF were in a group defined by boosting resistance. COPD is characterized by lung distension, cough, and phlegm/fluid retention in traditional Chinese medicine, and its basic pathogenesis is pulmonary retention of phlegm, pyrexia, and interlinking of phlegm and blood stasis [28, 29]. Furthermore, long periods of lung disease can cause lung* Qi* deficiency and lead to blood stasis and lung* Qi* stagnation. COPD may be alleviated by clearing heat, eliminating phlegm, activating blood circulation and stasis, invigorating* Qi*, and strengthening resistance. This was indeed the associated effect of the 12 included CHIs. The more details about the product information of 12 CHIs were shown in Table S6.

This study is the first to evaluate the efficacy and safety of CHIs for the treatment of AECOPD using a network meta-analysis. The clinical efficacy of included CHIs was evaluated on different outcomes, aiming to provide evidence and suggestions for the clinical selection of drugs. However, some limitations should not be overlooked. First, the results of this NMA were limited by the quality of the included RCTs. Only 24.52% (38/155) of the RCTs described the generation of a randomized sequence, and eight of them adopted a high risk of bias, due to grouping by the order of admission. Additionally, most of trials had inadequate allocation concealment and blinded information. Thus, the methodological quality of the enrolled studies was not high. Second, all the studies have been carried out and published in China, and data of clinical trials in other languages or other countries was absent. For this reason, we are unable to determine whether the results of this study are applicable to other ethnic groups. Third, the interventions of the majority of eligible studies were CHIs combined with WM versus WM; thus, there was a lack of head-to-head studies of direct comparisons between two CHIs with large samples.

## 4. Conclusions

In conclusion, based on this NMA, DH plus WM was found to be obviously superior to other interventions on improving RCE of AECOPD. Considering RCE, lung functions, blood gas analysis indices, and inflammatory markers synthetically, HQ plus WM and RDN plus WM showed a preferable improvement on patients with AECOPD. Based on the limitation of this NMA, our results should be confirmed by more multicenter, larger-sample, and head-to-head RCTs. The safety of CHIs should be strictly monitored.

## Figures and Tables

**Table 1:** Ranking of CHIs for different outcomes based on SUCRA.

Intervention		CKZ+WM	CXQ+WM	DH+WM	HQ+WM	RDN+WM	SF+WM	SM+WM	SMI+WM	TRQ+WM	XBJ+WM	XXN+WM	XYP+WM	WM
RCE	SUCRA(%)	32.4	69.4	85.5	76.7	54.5	19.1	59.5	39.5	55.2	51.6	67.8	38.8	0
	N	9	9	13	6	10	7	12	6	48*∗*	22	7	7	154
	Rank	11	3	1	2	7	12	5	9	6	8	4	10	13
FEV_1_%	SUCRA(%)	43.3	49.2	51.9	69	75.1	47.8	76.6	-	41.9	58.8	-	20.1	16.2
	N	3	2	1	2	4	4	6	-	7	4	-	2	35
	Rank	8	6	5	3	2	7	1	-	9	4	-	10	11
FEV_1_/FVC	SUCRA(%)	45.8	56.1	30.4	48.2	73.63	52.1	66.9	-	58.3	67.6	-	30.9	20.1
	N	1	1	1	1	4	4	5	-	9*∗*	7	-	2	34
	Rank	8	5	10	7	1	6	3	-	4	2	-	9	11
PaCO_2_	SUCRA(%)	32.8	49.2	-	59.1	75.2	50.7	54.9	83.6	43.4	35	48.7	51.3	16.1
	N	3	4	-	1	2	2	3	2	14	7	1	1	40
	Rank	11	7	-	3	2	6	4	1	9	10	8	5	12
PaO_2_	SUCRA(%)	45.2	45.9	-	60.9	71.9	47.4	51	56.1	49.3	30.9	73.8	53.2	14.4
	N	3	4	-	1	2	2	3	2	14	7	1	1	40
	Rank	10	9	-	3	2	8	6	4	7	11	1	5	12
pH	SUCRA(%)	-	40.3	-	-	70	49.3	58.4	-	51.3	48.4	61	-	21.3
	N	-	2	-	-	2	4	3	-	11	5	1	-	28
	Rank	-	7	-	-	1	5	3	-	4	6	2	-	8
CRP	SUCRA(%)	45.9	-	52.2	60.6	53	-	37	57.4	63.3	51.3	59.2	48.8	21.3
	N	1	-	1	1	4	-	1	3	10	11	1	3	36
	Rank	9	-	6	2	5	-	10	4	1	7	3	8	11
WBCC	SUCRA(%)	50.4	-	-	-	67	-	-	58	43.1	59.6	-	45.1	26.8
	N	1	-	-	-	1	-	-	1	8	7	-	2	20
	Rank	4	-	-	-	1	-	-	3	6	2	-	5	7
N%	SUCRA(%)	84.1	-	-	-	-	-	-	66.1	29.7	56.5	-	43.5	20.2
	N	1	-	-	-	-	-	-	1	5	6	-	2	15
	Rank	1	-	-	-	-	-	-	2	5	3	-	4	6

N, number of RCTs; *∗*, it includes a direct comparison, that is, TRQ combining with WM versus XYP combining with WM.

**Table 2:** Details of adverse drug reactions (ADRs)/ adverse drug events (ADEs).

	Experimental group	Control group	No grouping
TRQ+WM vs. WM	20 cases: Fever, 8 cases; pruritus, 3 cases; nausea, 2 cases; vomiting, 2 cases; nausea and vomiting, 2 cases, popular rash, 2 cases; diarrhea, 1 case	23 cases: Nausea and vomiting, 9 cases; dizziness, 4 cases; diarrhea, 4 cases; vomiting, 3 cases; nausea, 2 cases; chest pain, 1 case	10 cases: Pain due to fluid dropping too fast, 3 cases; nausea, 4 cases; dizziness, 1 case; headache, 1 case; loss of appetite, 1 case
XBJ+WM vs. WM	6 cases: Vasculitis, 3 cases; skin rash, 1 case; pruritus, 1 case; flushing, 1 case	0	/
DH+WM vs. WM	4 cases: Dizziness and flushing, 2 cases; elevated ALT, 2 cases	4 cases: Elevated ALT, 4 cases	/
SM+WM vs. WM	8 cases: Stimulation in oropharynx, 3 cases; palpitation, 2 cases; nystagmus, 2 cases; skin rash, 1 case	5 cases: Palpitation, 2 cases; nystagmus, 1 case; stimulation in oropharynx, 1 case; skin rash, 1 case	Mild headache, pruritus, skin rash and gastrointestinal adverse reactions
RDN+WM vs. WM	7 cases: Gastrointestinal adverse reactions, 3 cases; skin rash, 2 cases; dizziness, chest congestion and dry mouth, 2 cases	11 cases: Gastrointestinal adverse reactions, 6 cases; skin rash, 5 cases	/
CXQ+WM vs. WM	1 case: Minor allergy, 1 case	0	/
XYP+WM vs. WM	1 case: Diarrhea, 1 case	0	/
SF+WM vs. WM	1 case: Pruritus, 1 case	0	/
XXN+WM vs. WM	13 cases: Abdominal distension and nausea, 8 cases; dry mouth, 5 cases	0	/
SMI+WM vs. WM	5 cases: Gastrointestinal adverse reactions, 1 case; non-specific adverse reactions, 4 cases	5 cases: Gastrointestinal adverse reactions, 2 cases; non-specific adverse reactions, 3 cases	/
TRQ+WM vs. XYP+WM	0	2 cases: Phlebitis, 2 cases	/

## Abbreviations

**ADRs/ADEs:**: Adverse drug reactions/adverse drug events
**AECOPD:**: Acute exacerbation of chronic obstructive pulmonary disease
**CHI:**: Chinese herbal injection
**CIs:**: Confidence intervals
**CKZ:**: Chuankezhi injection
**CNKI:**: China National Knowledge Infrastructure Database
**COPD:**: Chronic obstructive pulmonary disease
**CRP:**: C-reaction protein
**CXQ:**: Chuanxiongqin injection
**DH:**: Danhong injection
**F****E****V**_1_%: The ratio of forced expiratory volume in the first second to the predicted value
**FEV**_1_**/FVC**: The ratio of forced expiratory volume in the first second to forced vital capacity
**HQ:**: Huangqi injection
**MD:**: Mean difference
N%: Neutrophil percentage
**NMA:**: Network meta-analysis
**ORs:**: Odds ratios
**PaCO**_2_: Arterial partial pressure of carbon dioxide
**PaO**_2_: Arterial partial pressure of oxygen
**RCE:**: Rate of clinical efficacy
**RCTs:**: Randomized controlled trials
**RDN:**: Reduning injection
**SF:**: Shenfu injection
**SM:**: Shenmai injection
**SMI:**: Shengmai injection
**SUCRA:**: Surface under the cumulative ranking probabilities
**TRQ:**: Tanreqing injection
**WBCC:**: White blood cell count
**WM:**: Western medicine
**XBJ:**: Xuebijing injection
**XXN:**: Xixinnao injection
**XYP:**: Xiyanping injection.

## Supplementary Materials

Supplementary 1 Table S1: PRISMA checklist for network meta-analysis.

Supplementary 2 Table S2: search strategy of PubMed.

Supplementary 3 Table S3: characteristics of included studies.

Supplementary 4 Table S4: network meta-analysis results of ORs and 95%CIs for rate of clinical effective.

Supplementary 5 Table S5: network meta-analysis results of MDs and 95%CIs for lung functions.

Supplementary 6 Table S6: more details about the product information of 12 CHIs.
