# Supplementary material for: Comparative Efficacy of Chinese Herbal Injections for Treating Acute Exacerbation of Chronic Obstructive Pulmonary Disease: A Bayesian Network Meta-Analysis of Randomized Controlled Trials
Source: Evid Based Complement Alternat Med. 2018 Jul 17;2018:7942936. doi: 10.1155/2018/7942936 (PMC6076913; doi:10.1155/2018/7942936)
Supplement: Supplementary 2 — Table S2: search strategy of PubMed. [file 7942936.f2.doc]

Table S2. Search strategy of PubMed

| #1 | chronic obstructive pulmonary disease [MeSH Terms] |
| --- | --- |
| #2 | chronic obstructive airway disease [Title/Abstract] |
| #3 | chronic obstructive lung disease [Title/Abstract] |
| #4 | chronic airflow obstruction [Title/Abstract] |
| #5 | COPD [Title/Abstract] |
| #6 | COAD [Title/Abstract] |
| #7 | #1 OR #2 OR #3 OR #4 OR #5 OR #6 |
| #8 | tanreqing [Title/Abstract] |
| #9 | xuebijing [Title/Abstract] |
| #10 | danhong [Title/Abstract] |
| #11 | shenmai [Title/Abstract] |
| #12 | reduning [Title/Abstract] |
| #13 | chuanxiongqin injection [Title/Abstract] |
| #14 | ligustrazin injection [Title/Abstract] |
| #15 | chuanxiongzine injection [Title/Abstract] |
| #16 | chuankezhi [Title/Abstract] |
| #17 | xiyanping [Title/Abstract] |
| #18 | shenfu [Title/Abstract] |
| #19 | xixinnao [Title/Abstract] |
| #20 | huangqi injection [Title/Abstract] |
| #21 | astragalus injection [Title/Abstract] |
| #22 | shengmai [Title/Abstract] |
| #23 | #8 OR #9 OR #10 OR #11 OR #12 OR #13 OR #14 OR #15 OR #16 OR #17 OR #18 OR #19 OR #20 OR #21 OR #22 |
| #24 | random [All Fields] |
| #25 | #7 AND #23 AND #24 |
