# Supplementary material for: Comparative Efficacy of Chinese Herbal Injections for Treating Acute Exacerbation of Chronic Obstructive Pulmonary Disease: A Bayesian Network Meta-Analysis of Randomized Controlled Trials
Source: Evid Based Complement Alternat Med. 2018 Jul 17;2018:7942936. doi: 10.1155/2018/7942936 (PMC6076913; doi:10.1155/2018/7942936)
Supplement: Supplementary 3 — Table S3: characteristics of included studies. [file 7942936.f3.doc]

Table S3. Characteristics of included studies

| Study ID | N(E/C) | Sex(M/F) | Age(min-max, x±d） | Intervention of E | Intervention of C | Course (d) | Risk of bias | Outcomes | ADRs/ADEs |
| --- | --- | --- | --- | --- | --- | --- | --- | --- | --- |
| Liu J 2017 [1] | 39/39 | 43/35 | E:61-79 67.21±4.62 C:60-82 69.36±4.81 | WM | WM+TRQ 20ml | 42 | Random | ①③④⑤⑥ | Not mentioned |
| Liang W 2017 [2] | 35/35 | 45/25 | E:55-73 62.34±5.52 C:56-75 61.76±5.59 | WM | WM+TRQ 20ml | 7 | Random | ① | No have |
| He X 2016 [3] | 35/35 | 56/14 | E:51-79 69.39±7.56 C:50-80 70.24±6.89 | WM | WM+TRQ 20ml | 7 | Random | ①⑥ | Not mentioned |
| Liu C 2016 [4] | 32/31 | 44/19 | E:64-82 71.2±7.0 C:64-78 70.8±6.2 | WM | WM+TRQ 20ml-40ml | 7 | Random number table | ① | Not mentioned |
| Wu NN 2016 [5] | 56/64 | 80/40 | E:52-78 67.5 C:54-77 66.9 | WM | WM+TRQ 20ml | 10 | Random number table | ①⑦⑧ | Detailed description |
| Liu Z 2015 [6] | 78/78 | 112/44 | E:54-82 62.3±7.1 C:56-86 63.1±7.4 | WM | WM+TRQ 20ml | 7 | Random number table | ①② | Not mentioned |
| Du Y 2015 [7] | 60/60 | 82/38 | E:67.54±10.66 C:66.38±11.34 | WM | WM+TRQ 20ml | 10 | Random | ① | Not mentioned |
| Zhao SH 2015 [8] | 40/40 | 48/32 | E:53-78 68.78±7.05 C:52-79 67.5±7.51 | WM | WM+TRQ 20ml | 14 | Random number table | ①②③ | Not mentioned |
| Pan Y 2015 [9] | 71/71 | 112/30 | E:65.17±9.24 C:63.51±11.26 | WM | WM+TRQ 20ml | 15 | Random | ①④⑤⑥ | Detailed description |
| Wang TB 2015 [10] | 46/46 | 53/39 | 41-73 59.26±2.68 | WM | WM+TRQ 20ml | 7 | Random number table | ① | Not mentioned |
| Li LL 2014 [11] | 38/38 | 42/34 | 56-78 66.5±5.2 | WM | WM+TRQ 20ml | 7 | Random | ①⑦⑧ | Detailed description |
| Liang W 2014 [12] | 30/30 | 35/25 | E:60-75 62.93±4.80 C:55-75 63.30±5.40 | WM | WM+TRQ 20ml | 7 | Random | ①⑦ | No have |
| Shi QH 2014 [13] | 15/15 | 17/13 | E:45-72 C:46-74 | WM | WM+TRQ 20ml | 5 | Random | ①② | Not mentioned |
| Hua WS 2014 [14] | 20/20 | 21/19 | E:55-80 C:50-75 | WM | WM+TRQ 20ml | 14 | Random | ① | Not mentioned |
| Qiao ZY 2013 [15] | 46/46 | 55/37 | E:58-85 66.7±6.9 C:59-83 66.1±6.2 | WM | WM+TRQ 20ml | 7 | Random | ①② | Not mentioned |
| Lu JS 2013 [16] | 30/30 | 37/23 | E:43-74 59.3±8.6 C:42-73 58.4±7.2 | WM | WM+TRQ 20ml | 10 | Random | ①④⑤⑥⑦⑧⑨ | Not mentioned |
| Li HJ 2013 [17] | 40/40 | 51/29 | E:52-85 70.6±5.9 C:54-80 69.5±5.92 | WM | WM+TRQ 20ml | 7 | Random | ①⑦ | Not mentioned |
| Ma HM 2013 [18] | 60/60 | 84/36 | E:55-80 61.10±7.21 C:52-76 61.12±12.35 | WM | WM+TRQ 20ml | 10 | Random | ①③④⑤⑥⑦ | Not mentioned |
| Shao YS 2012 [19] | 36/32 | 40/28 | E:46-94 C:52-86 | WM | WM+TRQ 20ml | 7 | Random | ① | Not mentioned |
| Huang HM 2012 [20] | 60/60 | 64/56 | E:66.2±12.3 C:65.4±13.5 | WM | WM+TRQ 20ml | 7~14 | Random | ① | Detailed description |
| Shi P 2012 [21] | 45/41 | 55/31 | E:65-80 69.4±5.7 C:65-79 68.5±7.1 | WM | WM+TRQ 20ml | 10 | Random | ① | No have |
| Long H 2012 [22] | 64/60 | 88/36 | E:40-83 61.10±7.21 C:52-72 61.12±12.35 | WM | WM+TRQ 20ml | 7 | Random | ①③④⑤⑥⑦ | Not mentioned |
| Liu JB 2012 [23] | 31/31 | 37/25 | E:61.2±11.5 C:59.5±11.4 | WM | WM+TRQ 20ml | 14 | Random | ①③ | Detailed description |
| Tang B 2012 [24] | 26/24 | 37/13 | E:66.30±10.46 C:64.20±12.68 | WM | WM+TRQ 20ml | 15 | Random | ① | No have |
| Wei SZ 2011 [25] | 40/40 | 44/36 | E:47-74 59.26±8.56 C:46-72 58.08±9.28 | WM | WM+TRQ 20ml | 14 | Random | ①③ | Not mentioned |
| Zhou XM 2011 [26] | 60/60 | 75/45 | E:49-82 62.2±6.7 C:50-81 62.6±7.2 | WM | WM+TRQ 20ml | 10 | Random | ①②③④⑤⑥ | No have |
| Li CA 2010 [27] | 24/24 | 33/15 | E:48-73 60.1±7.6 C:45-72 58.2±8.3 | WM | WM+TRQ 20ml | 14 | Random | ① | Not mentioned |
| Fu Q 2009 [28] | 50/50 | 92/8 | E:80-93 85.72 C:81-94 86.58 | WM | WM+TRQ 20ml | 7~10 | Random | ①② | Not mentioned |
| Ma L 2009 [29] | 30/30 | 50/10 | 60-86 | WM | WM+TRQ 20ml | 14 | Random | ① | Not mentioned |
| Zha SQ 2009 [30] | 30/30 | 34/26 | E:65-75 69.3±5.6 C:65-79 67.8±7.2 | WM | WM+TRQ 20ml | 14 | Random | ① | Not mentioned |
| Dong YH 2009 [31] | 36/36 | 47/25 | E:45-93 64.8±13.6 C:47-89 63.4±12.9 | WM | WM+TRQ 20ml | 10~14 | Random | ①⑦ | Not mentioned |
| Luo GW 2009 [32] | 35/32 | 53/14 | E:54-78 68.2±6.5 C:50-76 67.3±6.7 | WM | WM+TRQ 20ml | 14 | Random | ①⑦ | Not mentioned |
| Zhao KP 2009 [33] | 44/40 | 46/38 | E:24-75 61.49±4.87 C:25-74 60.32±4.69 | WM | WM+TRQ 20ml | 10 | Random | ① | Not mentioned |
| Fu YW 2009 [34] | 65/63 | 68/60 | E:43-77 61.2±1.2 C:47-77 61.8±2.8 | WM | WM+TRQ 20ml | 10 | Random | ①④⑤ | No have |
| Zhou Z 2009 [35] | 72/50 | 83/39 | E:40-85 63.67±7.25 C:43-72 65.27±9.35 | WM | WM+TRQ 20ml | 10~14 | Random | ①④⑤⑥ | No have |
| Yin LB 2009 [36] | 32/30 | 44/18 | E:52-85 73.38±7.61 C:54-85 74.21±10.47 | WM | WM+TRQ 20ml | 10 | Random | ①④⑤⑥⑦⑧⑨ | No have |
| Cui ML 2009 [37] | 46/44 | 62/28 | E:69.0±7.1 C:70.3±7.3 | WM | WM+TRQ 20ml | 12 | Random | ①⑧ | No have |
| Zhou J 2008 [38] | 43/38 | 43/38 | E:68-75 69.3±5.6 C:65-76 67.8±7.2 | WM | WM+TRQ 20ml | 14 | Random | ① | Detailed description |
| Yang YH 2008 [39] | 30/30 | 53/7 | E:63.2±4.8 C:65.2±8.4 | WM | WM+TRQ 20ml | 10 | Random | ①②③④⑤ | Detailed description |
| Li X 2008 [40] | 40/40 | 52/28 | 43-82 63.21±7.26 | WM | WM+TRQ 20ml | 10 | Random | ①④⑤⑥ | Not mentioned |
| Wang CC 2008 [41] | 24/24 | 35/13 | E:61-89 76.29±6.21  C:56-90 72.21±8.18 | WM | WM+TRQ 20ml | 7 | Random | ①④⑤⑧⑨ | Detailed description |
| Liu ZX 2007 [42] | 100/98 | 103/95 | E:43-77 61.2±1.2 C:47-77 61.8±2.8 | WM | WM+TRQ 20ml | 7 | Random | ① | Not mentioned |
| Mao XM 2007 [43] | 79/88 | 108/59 | E:48-78 63.9 C:49-75 63.2 | WM | WM+TRQ 20ml | 10 | Random | ①④⑤⑧⑨ | No have |
| Wang SQ 2006 [44] | 66/52 | 82/36 | 56-78 68 | WM | WM+TRQ 20ml | 21 | Random | ① | Detailed description |
| Zhang W 2006 [45] | 37/37 | 43/31 | E:63.25±10.15 C:62.50±11.30 | WM | WM+TRQ 20ml | 7 | Random | ①②④⑤ | No have |
| Xie YH 2005 [46] | 52/30 | 49/33 | E:45-78 63.67±8.21 C:52-72 63.41±9.35 | WM | WM+TRQ 20ml | 15 | Random | ①⑥ | No have |
| Zhang Y 2004 [47] | 29/29 | 38/20 | E:71.48±7.72 C:69.34±7.83 | WM | WM+TRQ 20ml | 12 | Random number table, single-blind, allocation concealment | ①⑧⑨ | Detailed description |
| Xi R 2017 [48] | 39/39 | 27/51 | E:65.44±1.44 C:65.48±1.89 | WM | WM+XBJ 100ml | 10 | Random | ①⑦⑧ | No have |
| Xu LM 2017 [49] | 53/53 | 62/44 | 48.49±4.87 | WM | WM+XBJ 100ml | 7 | Random number table | ①⑦⑧⑨ | Not mentioned |
| Li Y 2016 [50] | 41/41 | 43/39 | E:69.3±7.2 C:70.3±6.8 | WM | WM+XBJ 100ml | 14 | Random number table | ①③⑦⑧⑨ | Not mentioned |
| Yao LF 2016 [51] | 30/30 | 33/27 | E:64.8±2.5 C:65.5±2.2 | WM | WM+XBJ 100ml | 21 | Random number table | ① | Not mentioned |
| Zhu J 2016 [52] | 38/38 | 51/25 | E:62.54±10.57 C:63.07±9.88 | WM | WM+XBJ 50ml | 8 | Random | ①⑦ | Not mentioned |
| Duan LM 2015 [53] | 46/46 | 52/40 | 70.3±5.8 | WM | WM+XBJ 100ml | 7 | Random | ① | No have |
| Wang XQ 2015 [54] | 30/30 | 29/31 | E:69.6 C:71.0 | WM | WM+XBJ 100ml | 7 | Random | ①⑦⑧⑨ | No have |
| Zhang H 2015 [55] | 50/50 | 78/22 | E:71.00±10.00 C:70.00±9.00 | WM | WM+XBJ 100ml | 7 | Random | ①④⑤⑦ | Not mentioned |
| Wang N 2014 [56] | 32/31 | 36/27 | E:67.4±4.6 C:69.2±5.8 | WM | WM+XBJ 100ml | 7 | Random number table | ①④⑤⑥ | Not mentioned |
| Mu L 20145 [57] | 36/36 | 43/29 | 51-82 | WM | WM+XBJ 100ml | 7 | Random | ①④⑤⑥⑦⑧⑨ | Not mentioned |
| Fu HW 2014 [58] | 30/27 | 38/19 | E:58.7±5.8 C:59.2±5.4 | WM | WM+XBJ 80ml | 5 | Random | ①③④⑤⑥ | Not mentioned |
| Shao N 2013 [59] | 30/30 | 50/10 | E:59.1±16.2 C:58.2±17.2 | WM | WM+XBJ 200ml | 9 | Random | ①⑦⑧⑨ | Not mentioned |
| Zhao L 2013 [60] | 36/36 | 42/30 | E:69.2±9.4 C:71.2±8.3 | WM | WM+XBJ 100ml | 10 | Random | ① | Detailed description |
| Qiu Q 2013 [61] | 35/35 | 48/22 | 72.4±11.3 | WM | WM+XBJ 200ml | 10 | Random | ①③ | Not mentioned |
| Ni XH 2012 [62] | 32/28 | 31/29 | E:65.1±7.0 C:64.8±6.8 | WM | WM+XBJ 100ml | 7 | Random | ①③④⑤⑥ | Not mentioned |
| Fan DB 2012 [63] | 86/86 | 97/75 | E:60.48±8.34 C:60.25±8.36 | WM | WM+XBJ 50ml | 14 | Random | ①②③ | No have |
| Li Q 2012 [64] | 60/60 | 79/41 | E:41-79 C:45-76 | WM | WM+XBJ 100ml | 10 | Random | ①②③⑦ | Not mentioned |
| Hao TP 2010 [65] | 30/30 | 42/18 | E:66±6 C:67±5 | WM | WM+XBJ 100ml | 10 | Random | ①⑦ | Not mentioned |
| Tu SY 2010 [66] | 24/24 | 41/7 | E:70.54±10.62 C:69.08±9.13 | WM | WM+XBJ 100ml | 7 | Random | ①④⑤⑥⑦⑧⑨ | Detailed description |
| Lin DB 2009 [67] | 30/30 | 44/16 | E:71.5 C:70.8 | WM | WM+XBJ 100ml | 7 | Random | ① | Not mentioned |
| Ye L 2009 [68] | 30/27 | 50/7 | E:75.3±9.2 C:71.6±10.3 | WM | WM+XBJ 100ml | 10 | Order of admission, single-blind | ①②③④⑤ | No have |
| Wang P 2007 [69] | 65/61 | 85/41 | E:49-88 C:47-85 | WM | WM+XBJ 100ml | 10 | Random | ① | Detailed description |
| Jia ZR 2017 [70] | 62/62 | 73/51 | E:63.5±5.8 C:62.7±6.1 | WM | WM+DH 20ml | 7-10 | Random | ① | Not mentioned |
| Ge ZK 2016 [71] | 32/32 | 41/23 | E:54.9±4.4 C:55.7±4.6 | WM | WM+DH 20ml | 14 | Random | ① | Not mentioned |
| Du J 2014 [72] | 30/30 | 34/26 | E:65±8.4 C:62±9.8 | WM | WM+DH 30ml | 14 | Random | ① | Not mentioned |
| Zhang Q 2014 [73] | 64/64 | 71/57 | E:64.45±9.46 C:64.41±9.45 | WM | WM+DH 40ml | 14 | Dicing | ① | No have |
| Chen Z 2013 [74] | 52/48 | 60/40 | E:66.6±7.4 C:63.8±7.8 | WM | WM+DH 40ml | 14 | Random number table | ① | Not mentioned |
| Wang JH 20135 [75] | 31/31 | 46/16 | E:76.35±5.7 C:69.28±5.18 | WM | WM+DH 30ml | 14 | Random | ① | Not mentioned |
| Zhang L 2013 [76] | 60/60 | 56/64 | 70.6±3.3 | WM | WM+DH 20ml | 14 | Order of admission | ① | Not mentioned |
| Wang H 2012 [77] | 40/40 | 42/38 | E:61.2±12.5 C:65.3±12.7 | WM | WM+DH 20ml | 14 | Random number table | ①⑦ | Detailed description |
| Liu Z 2010 [78] | 50/46 | 62/34 | E:67.7±9.8 C:66.1±9.2 | WM | WM+DH 40ml | 14 | Random number table | ① | Not mentioned |
| Yang RF 2010 [79] | 42/40 | 63/19 | E:66.5±5.8 C:67.2±4.6 | WM | WM+DH 20ml | 14 | Random | ① | Not mentioned |
| Shi YY 2009 [80] | 23/23 | 27/19 | E:58 C:57 | WM | WM+DH 20-30ml | 14 | Random | ①②③ | Not mentioned |
| Tang TZ 2009 [81] | 68/54 | 74/48 | E:68 C:62 | WM | WM+DH 40ml | 14 | Order of admission | ① | Detailed description |
| Chen HC 2007 [82] | 40/36 | 60/16 | E:78.45 C:77.69 | WM | WM+DH 40ml | 14 | Random number table | ① | No have |
| Shen F 2017 [83] | 65/65 | 77/53 | E:63.4±5.6 C:62.8±5.9 | WM | WM+SM 100ml | 14 | Random | ① | Not mentioned |
| Ran ZW 2017 [84] | 64/64 | 75/53 | E:63.4±5.6 C:62.8±5.9 | WM | WM+SM 40ml | 14 | Random number table | ①③⑦ | Not mentioned |
| Luo XE 2016 [85] | 56/56 | 62/50 | 61.6±9.7 | WM | WM+SM 100ml | 14 | Order of admission | ①②③④⑤⑥ | Detailed description |
| Xie XX 2016 [86] | 33/32 | 45/20 | …… | WM | WM+SM 40ml | 10-14 | Random | ①②③ | Detailed description |
| Yang JW 2015 [87] | 60/60 | 82/38 | E:69±5.2 C:70±5.5 | WM | WM+SM 100ml | 14 | Order of admission | ①②③④⑤⑥ | No have |
| Haung XL 2014 [88] | 50/50 | 58/42 | E:69.32±5.74 C:70.45±7.55 | WM | WM+SM 50ml | 7 | Random | ①②④⑤⑥ | Not mentioned |
| Yin ZH 2013 [89] | 110/114 | 134/90 | E:59.15 C:60.11 | WM | WM+SM 60ml | 7-14 | Random | ①② | No have |
| Li Y 2013 [90] | 43/43 | 46/40 | E:46-94 C:52-86 | WM | WM+SM 60ml | 10 | Random | ① | Not mentioned |
| Li C 2010 [91] | 28/27 | …… | …… | WM | WM+SM 40ml | 14 | Random | ①②③ | Not mentioned |
| Guo X 2009 [92] | 35/35 | 40/30 | E:67 C:66 | WM | WM+SM 40ml | 15 | Random | ① | Not mentioned |
| Yang HZ 2005 [93] | 30/30 | 38/22 | E:64.5 C:62.6 | WM | WM+SM 30ml | 15 | Random | ① | No have |
| Li JL 2001 [94] | 42/34 | 41/35 | E:57.5 C:56 | WM | WM+SM 40ml | 10 | Random | ① | Not mentioned |
| Ye SH 2016 [95] | 40/40 | 45/35 | E:42-75 62.4±3.9 C:41-77 62.8±3.4 | WM | WM+RDN 20mL | 28-35d | Random number table | ①②③ | Not mentioned |
| Dong HZ 2015 [96] | 30/30 | 39/21 | E:45-83 62.3±1.9 C:41-80 61.3±2.1 | WM | WM+RDN 20mL | 7d | Random number table | ①②④ | Not mentioned |
| Tian TL 2015 [97] | 37/37 | 42/32 | E:59.7 C:60.8 | WM | WM+RDN 20mL | 10d | Random | ①②③ | Detailed description |
| Zhou JG 2014 [98] | 30/30 | 32/28 | E:58-84 72.6±5.4 C:56-85 73.1±5.8 | WM | WM+RDN 20mL | 7d | Random | ①⑤⑥⑦ | Detailed description |
| Sun GX 2012 [99] | 52/50 | 58/44 | M:64.56±10.47 F:65.62±10.85 | WM | WM+RDN 20mL | 7-10d | Random | ①②③④⑤⑥⑦⑧ | No have |
| Yao SX 2012 [100] | 50/50 | 55/45 | E:49-80 62.1±6.8 C:50-81 62.4±7.3 | WM | WM+RDN 20mL | 7d | Random | ①②③ | No have |
| Zhan XL 2012 [101] | 30/30 | 40/20 | 60-82 | WM | WM+RDN 20mL | 7-10d | Random | ① | Not mentioned |
| Yue YX 2014 [102] | 33/32 | 41/24 | E:61-82 73±4.8 C:60-80 70±4.5 | WM | WM+RDN 20mL | 7-14d | Random | ①⑦ | Not mentioned |
| Zhsng N 2011 [103] | 45/42 | 67/20 | E:56-72 66.2±5.5 C:53-70 65.7±5.6 | WM | WM+RDN 20mL | 10d | Random | ①⑦ | Not mentioned |
| Chen YB 2017 [104] | 60/60 | 68/52 | E:45-75 65±2.1 C:40-80 60±2.5 | WM | WM+RDN 20mL | 7d | Random | ① | Not mentioned |
| Chen XD 2014 [105] | 43/43 | 62/24 | E:68.8±7.2 C:68.1±6.9 | WM | WM+CXQ 120mg | 10 | Random number table | ①②③ | No have |
| Liu Y 2014 [106] | 60/60 | 82/38 | E:67.7±7.1 C:68.1±7.2 | WM | WM+CXQ 120mg | 10 | Random number table | ①④⑤⑥ | No have |
| Wang ZF 2013 [107] | 30/30 | 44/16 | 64.3 | WM | WM+CXQ 40mg | 14 | Order of admission | ① | Not mentioned |
| Wu BM 2013 [108] | 98/90 | 108/80 | …… | WM | WM+CXQ 120mg | 12 | Random | ① | Detailed description |
| Li Q 2010 [109] | 48/48 | 67/29 | E:64.7±11.2 C:63.8±11.5 | WM | WM+CXQ 200mg | 10-14 | Random | ① | No have |
| Wu NN 2008 [110] | 30/30 | 35/25 | E:65.32±11.56 C:61.56±10.23 | WM | WM+CXQ 100mg | 14 | Random number table | ①④⑤ | No have |
| Ke JY 2008 [111] | 54/52 | 59/47 | 65.3±5.8 | WM | WM+CXQ 20-40ml | 10-14 | Random | ①② | Not mentioned |
| Li ZM 2007 [112] | 25/25 | 38/12 | E:69.8±4.62 C:68.4±3.56 | WM | WM+CXQ 160mg | 7 | Random | ①④⑤ | Not mentioned |
| Haung MH 2004 [113] | 26/15 | 29/12 | E:63.5±8.2 C:65.1±7.9 | WM | WM+CXQ 160mg | 14 | Random | ①②④⑤⑥ | Not mentioned |
| Wang HS 2017 [114] | 80/80 | 91/69 | E:61.43±8.95 C:59.63±9.31 | WM | WM+CKZ 4ml | 14 | Random number table | ①③④⑤ | Not mentioned |
| Song L 2012 [115] | 40/40 | 58/22 | E:52-82 C:54-84 | WM | WM+CKZ 4ml | 7 | Random | ① | Not mentioned |
| Liu YK 2010 [116] | 40/40 | 75/5 | E:52-91 C:50-88 | WM | WM+CKZ 4ml | 15 | Random | ①④⑤⑦⑧⑨ | Not mentioned |
| Cui YD 2010 [117] | 24/24 | 31/17 | E:50-74 C:51-76 | WM | WM+CKZ 4ml | 14 | Random | ① | No have |
| Liao HL 2008 [118] | 20/20 | 24/16 | E:62.4 C:64.6 | WM | WM+CKZ 4ml | 14 | Random | ①② | No have |
| Wang DP 2014 [119] | 37/37 | 38/36 | 60.82±2.92 | WM | WM+CKZ 4ml | 15 | Random number table | ① | Not mentioned |
| Yuan GH 2014 [120] | 39/39 | 22/56 | E:74.4±5.1 C:74.6±4.7 | WM | WM+CKZ 4ml | 21 | Random number table | ①④⑤ | Not mentioned |
| Zhong RD 2012 [121] | 30/30 | 44/16 | E:81±4.5 C:82±5.3 | WM | WM+CKZ 4ml | 14 | Random | ① | No have |
| Qian P 2015 [122] | 32/32 | 39/25 | E:60.58±3.23 C:59.98±3.37 | WM | WM+CKZ 4ml | 14 | Random number table | ① | Not mentioned |
| Han DL 2012 [123] | 23/27 | 32/18 | E:46-75 C:48-76 | WM | WM+XYY 8ml | 14 | Random | ①②⑦ | Not mentioned |
| Zhang X 2014 [124] | 46/46 | 62/30 | E:65-84 67.8±7.4 C:65-82 67.6±7.2 | WM | WM+XYY 250mg | 14 | Random number table | ①③⑦ | Detailed description |
| Wu DJ 2015 [125] | 40/40 | 72/8 | 53-84 65.29±3.48 | WM | WM+XYY 250mg | 14 | Random number table | ① | Not mentioned |
| Jiang XF 2016 [126] | 49/48 | 53/44 | E:52-79 61.9±4.3 C:51-78 62.1±4.5 | WM | WM+XYY 250mg | 14 | Random | ①③ | Not mentioned |
| Zahng WJ 2011 [127] | 40/40 | 46/34 | E:65-77 68.3±5.5 C:65-79 66.9±7.4 | WM | WM+XYY 8ml | 10 | Random | ①⑦⑧⑨ | No have |
| Qian YR 2013 [128] | 30/30 | 36/24 | E:52-65 C:55-70 | WM | WM+XYY 250mg | 10 | Random | ①②④⑤⑧⑨ | No have |
| Zhang YL 2017 [129] | 39/39 | 47/31 | E:64.3±14.7 C:65.1±14.9 | WM | WM+SF 50ml | 14 | Random | ①②③⑥ | No have |
| Chi YS 2015 [130] | 48/48 | 42/54 | E:76.45±5.66 C:77.68±6.21 | WM | WM+SF 50ml | 14 | Random number table | ①④⑤⑥ | Detailed description |
| Han BY 2013 [131] | 36/36 | 42/30 | 62.34±2.27 | WM | WM+SF 100ml | 14 | Random number table | ①②③④⑤⑥ | No have |
| Ren YJ 2013 [132] | 35/35 | 41/29 | E:62.5±5.4 C:62.8±5.1 | WM | WM+SF 50ml | 14 | Random | ① | Not mentioned |
| Xu GL 2012 [133] | 37/30 | 60/7 | …… | WM | WM+SF 50ml | 10 | Order of admission | ① | Not mentioned |
| Qin HJ 2010 [134] | 35/35 | 47/23 | E:60.5±11.5 C:61.3±10.8 | WM | WM+SF 50ml | 7 | Random | ①②⑥ | Not mentioned |
| Liao WS 2008 [135] | 30/28 | 39/19 | E:68.3±7.4 C:65.2±5.9 | WM | WM+SF 50ml | 14 | Random | ①②③ | Not mentioned |
| Li ST 2017 [136] | 35/35 | …… | …… | WM | WM+XXN 24mg | 10 | Random | ① | Not mentioned |
| Lu N 2015 [137] | 48/48 | 42/54 | E:65.9±4.8 C:64.2±5.0 | WM | WM+XXN 16-24mg | 14 | Random | ① | Not mentioned |
| Ye Q 2015 [138] | 45/45 | 67/23 | 62±8 | WM | WM+XXN 24mg | 7 | Random | ①④⑤⑥⑦ | Not mentioned |
| Zhou HY 2009 [139] | 40/40 | 50/30 | 60-90 | WM | WM+XXN 24mg | 14 | Random | ① | Not mentioned |
| Dai XZ 2008 [140] | 34/24 | 37/21 | 64 | WM | WM+XXN 16mg | 5-7 | Order of admission | ① | Not mentioned |
| Dong GB 2008 [141] | 120/120 | 138/102 | E:62.5 C:61.2 | WM | WM+XXN 24mg | 7-14 | Random | ① | Detailed description |
| Zhang ZH 2006 [142] | 44/44 | 66/22 | E:62.21±11.21 C:61.32±12.35 | WM | WM+XXN 16-24mg | 15 | Random | ① | No have |
| Xiong SQ 2013 [143] | 56/56 | 63/49 | E:66.7 C:66.5 | WM | WM+HQ 40ml | 14 | Random | ①②③⑤ | Not mentioned |
| Zhang CM 2014 [144] | 30/28 | 35/23 | E:65.1±5.4 C:63.8±5.1 | WM | WM+HQ 30ml | 14 | Random | ① | Not mentioned |
| Tang W 2012 [145] | 56/56 | 65/47 | E:72.5±5.0 C:71.0±4.2 | WM | WM+HQ 40ml | 21 | Random | ① | Not mentioned |
| Zhang T 2012 [146] | 26/25 | 40/11 | E:61±11 C:66±10.3 | WM | WM+HQ 20ml | 10 | Random | ①⑦ | Not mentioned |
| Luo XB 2008 [147] | 20/25 | 37/8 | E:62±12 C:64±10 | WM | WM+HQ 60ml | 10 | Random | ①② | Not mentioned |
| Zhang C 2000 [148] | 36/36 | 37/35 | E:41-85 C:40-82 | WM | WM+HQ 20ml | 14 | Random | ①④ | Not mentioned |
| Zhou Q 2015 [149] | 31/31 | 37/25 | E:64.63±7.32 C:63.57±6.73 | WM | WM+SMI 50ml | 14 | Random | ① | Not mentioned |
| Chen AZ 2013 [150] | 31/31 | 37/25 | 61.4±5.6 | WM | WM+SMI 40ml | 7 | Random | ①⑦ | Detailed description |
| Wang Y 2007 [151] | 32/28 | 51/9 | E:69.5±7.8 C:69.3±8.0 | WM | WM+SMI 60ml | 14 | Random | ①④⑤⑧⑨ | No have |
| Gao XL 2006 [152] | 25/20 | 33/12 | E:60.46±9.58 C:60.52±8.65 | WM | WM+SMI 100ml | 7 | Random | ①⑦ | Not mentioned |
| Lv R 2015 [153] | 36/36 | 45/27 | 70.57±5.76 | WM | WM+SMI 50ml | 7 | Random | ①④⑤⑦ | Detailed description |
| Chen HH 2008 [154] | 45/40 | 46/39 | E:63-90 C:64-92 | WM | WM+SMI 30ml | 14 | Random | ① | No have |
| Zhang ZG 2017 [155] | 68/68 | 91/45 | E:68.5±7.2；C：70.3±6.3 | WM+TRQ 20ml | WM+XYP 150ml | 10 | Random number table | ①③ | Detailed description |

Note: N, number; E, experimental; C, control; M, male; F, female; d, day; TRQ, Tanreqing injection; XBJ, Xuebijing injection; DH, Danhong injection; SM, Shenmai injection; RDN, Reduning injection; CXQ, Chuanxiongqin injection; CKZ, Chuankezhi injection; XYP, Xiyanping injection; SF, Shenfu injection; XXN, Xixinnao injection; HQ, Huangqi injection; SMI, Shengmai injection; ①, rate of clinical efficacy; ②, ratio of force expiratory volume in the first second to predicted value; ③, ratio of force expiratory volume in the first second to forced vital capacity; ④, arterial partial pressure of carbon dioxide; ⑤, arterial partial pressure of oxygen; ⑥, blood's pH; ⑦, C-reaction protein; ⑧, white blood cell count ; ⑨, neutrophil percentage; ADRs, adverse drug reactions; ADEs, adverse drug events; WM, Western Medicine. WM contained inhaled bronchodilators (such as beta-agonists, anti-muscarinic agents, theophylline drugs), expectorants and anti-inflammatory agents.

References of included studies

1 Liu J. Clinical observation on Tanreqing Injection combined with low molecular weight heparin in treatment of elderly COPD in acute exacerbation. Clin Med. 2017; 39: 57.

2 Liang W, Chen SN, Li RX. Effect of hs-CRP, IL-6 and IL-10 and clinical research on efficacy of cute exacerbation of Chronic Obstructive Pulmonary Disease treating with Tanreqing Injection. J Guangxi Univ Tradit Chin Med. 2017;20: 11-3.

3 He X. Clinical observation on Tanreqing injection combined with conventional western medicine in treatment of Chronic Obstructive Pulmonary Disease in acute exacerbation. J Hubei Univ Tradit Chin Med. 2016;18: 74-6.

4 Li C. Clinical Observation on Tanreqing Injection in treatment of exacerbation of Chronic Obstructive Pulmonary Disease. China Health Nutr. 2016;26: 19-20.

5 Wu NN, Zhang Y, Li MX. Clinical observation of exacerbation of Chronic Obstructive Pulmonary Disease with the Tanreqing Injection. Chin J of Clinl Ration Drug Use. 2016;9: 80-2.

6 Liu Z, Zhang YL. Treating 78 casese of exacerbation of COPD with the Tan’re Qing Injection. Clin J Tradit Chin Med. 2015;7: 83-5.

7 Du Y, Jie ZJ, He W. Study of influence of Tanreqing Injection on serum high-sensitivity C-reactive protein and protein and procalcitionin of patients with acute exacerbation of Chronic Obstructive Pulmonary Disease. Chin J Nosocomiol. 2015; 25: 1233-35.

8 Zhao SH. Clinical observation of Tanreqing Injection on acute exacerbation of COPD. J Emerg Syndromes Tradit Chin Med. 2015;24: 1467-8.

9 Pan Y, Fu XH, Gao JZ, Wang ML. Effect of Serum cytokines and clinical research on efficacy of cute exacerbation of Chronic Obstructive Pulmonary Disease treating with Tanreqing Injection combined with basic therapy. Guiding J Tradit Chin Med Pharm. 2015;21: 66-8.

10 Wang TB. Effects of Tanreqing Injection on acute exacerbations of Chronic Obstructive Pulmonary Disease. Chin J Pract Med. 2015;42: 32-3.

11 Li LL, Xue QL, Xiang BL, Liu YF, Han SC. Clinical observation of Tanreqing Injection on acute exacerbation of Chronic Obstructive Pulmonary Disease. J Ningxia Med Univ. 2014;36: 204-6.

12 Liang W. Tanreqing injection for COPD patients with acute aggravating period of IL-6 and IL-10. Guangxi Univ Chin Med. 2014.

13 Shi QH, Ma X, Yang C. Clinical observation of exacerbation of Chronic Obstructive Pulmonary Disease with the Tanreqing Injection. Med Inf. 2014;27: 479.

14 Hua WS. Treating 78 casese of AECOPD with the Tanreqing Injection. Hunan J Tradit Chin Med. 2014;30: 32-3.

15 Qiao ZY. Treating 46 casese of AECOPD with the Tanreqing Injection. Natl Med Front Chin. 2013; 8: 27.

16 Lu JS, Li Q, Zhao WX, Zheng HY, Zhang FH, Lian B. Study of influence of Tanreqing Injection on blood-gas analysis , blood routine and C-Reactive Protein of patients with AECOPD. Clin J Tradit Chin Med. 2013;25: 29-30.

17 Li HJ. Clinical observation of Tanreqing Injection on AECOPD. J Emerg Syndromes Tradit Chin Med. 2013;22: 1021.

18 Ma HM, Xing YP, Xu F. Clinical observation of Tanreqing Injection on acute exacerbation of Chronic Obstructive Pulmonary Disease. For all Health 2013;7: 29-30.

19 Shao YS. Clinical observation on Tanreqing injection combined with antibiotics in treatment of Chronic Obstructive Pulmonary Disease in acute exacerbation. Chin Primary Health Care. 2012;26: 113.

20 Huang HM. Clinical observation on Tanreqing Injection combined with Levofloxacin in treatment of COPD in acute exacerbation. Strait Pharm J. 2012;24: 168-9.

21 Shi P. Clinical observation of Tanreqing Injection on elderly AECOPD. J Emerg Syndromes Tradit Chin Med. 2012;21: 1140-1.

22 Long H. Treating 124 casese of AECOPD with the Tanreqing Injection. J Emerg Syndromes Tradit Chin Med. 2012;21: 966.

23 Liu JB. Observation on Tanreqing Injection in treatment of Chronic Obstructive Pulmonary Disease. Pharmacol Clin Chin Mater Med. 2012;28: 170-2.

24 Tang B, Wang ZX, Xie HQ, Ye Y. Observation on Tanreqing Injection in treatment of Chronic Obstructive Pulmonary Disease in acute exacerbation. J Emerg Syndromes Tradit Chin Med. 2012;21: 1345-6.

25 Wei SZ, Chen SN, Feng Y. Study of influence of Tanreqing Injection on cytokines and lung function of patients with acute exacerbation of Chronic Obstructive Pulmonary Disease. J Emerg Syndromes Tradit Chin Med. 2011;20: 1402-3.

26 Zou XM, Dong YR, Zhang CL. Clinical observation on Tanreqing Injection in treatment of COPD in acute exacerbation. Mod J Integr Tradit Chin West Med. 2011;20: 3957-8.

27 Li CA. Study of influence of Tanreqing Injection on CRP of patients with acute exacerbation of Chronic Obstructive Pulmonary Disease. Guangming Tradit Chin Med. 2010;25: 999-1000.

28 Fu Q, Li L, Tan F. Experience of Tanreqing Injection with antibiotics on elderly AECOPD. J Emerg Syndromes Tradit Chin Med. 2009;18: 120-1.

29 Ma L, Li XD, Li CH. Clinical observation on Tanreqing injection combined with piperacillin-tazobactam in treatment of elderly COPD in acute exacerbation. J Emerg Syndromes Tradit Chin Med. 2009;18: 338.

30 Zha SQ. Clinical observation on Tanreqing injection combined with Cefoperazone-sulbactam in treatment of elderly COPD in acute exacerbation. Chin Med Herald. 2009;6: 98.

31 Dong YH. Clinical observation of Tanreqing Injection on acute exacerbation of Chronic Obstructive Pulmonary Disease. J Snake.2009;21: 283-4.

32 Luo GW, Sun JM, Mao XM. Study of influence of Tanreqing Injection on C-Reactive Protein of patients with acute exacerbation of Chronic Obstructive Pulmonary Disease. J Emerg Syndromes Tradit Chin Med. 2009;18: 1954-5.

33 Zhao KM, Xu YL, Qu NN. Efficacy of Tanreqing Injection combined with antibiotics in treating acute exacerbation of Chronic Obstructive Pulmonary Disease. J Liaoning Univ Tradit Chin Med. 2009;11: 127-8.

34 Fu YW. Clinical observation of Tanreqing Injection on acute exacerbation of Chronic Obstructive Pulmonary Disease. Chin Med Herald. 2009;6: 147.

35 Zhou Z, Wen CX. Clinical observation on Tanreqing Injection in treatment of COPD in acute exacerbation. J Emerg Syndromes Tradit Chin Med. 2009;18: 5-7.

36 Yin LB. Treating 32 casese of acute exacerbation of Chronic Obstructive Pulmonary Disease with the Tanreqing Injection. Jiangxi J Tradit Chin Med. 2009;40: 25-6.

37 Cui ML, Jia PM, Liu CL. Clinical observation of Tanreqing Injection on acute exacerbation of Chronic Obstructive Pulmonary Disease. J Changzhi Med Coll. 2009;23: 372-4.

38 Zhou J, Wu B, Cai FB. Observation of Tanreqing Injection combined with lomefloxacin in treating acute exacerbation of Chronic Obstructive Pulmonary Disease. Chin Foreign Med Treat. 2008;23: 73.

39 Yang YH. Effect of IL-8 in the peripheral blood and clinical research on efficacy of cute exacerbation of Chronic Obstructive Pulmonary Disease treating with Tanreqing Injection. Fujian Coll of Chin Med. 2008.

40 Li X, Huang HQ, Zhang CL. Clinical observation on Tanreqing injection combined with Western medicine in treatment of Chronic Obstructive Pulmonary Disease in acute exacerbation. Chin J Mod Drug Appl.2008;2: 83-4.

41 Wang CC. Clinical observation on acute exacerbation of Chronic Obstructive Pulmonary Disease treating with Tanreqing Injection. Zhejiang Univ Chin Med. 2008.

42 Liu ZX, Wei AM. Effect of Tanreqing Injection in treatment of 100 paties with the exacerbation of Chronic Obstructive Pulmonary Disease. Cent Plains Med J. 2007;34: 27-8.

43 Mao XM, Sun JM, Luo GW. Clinical observation of Tanreqing Injection on acute exacerbation of Chronic Obstructive Pulmonary Disease. J Clin Pulm Med. 2007;12: 641-2.

44 Wang SQ, Gao P. Effect of Tanreqing Injection combined with antibiotics in treatment of 66 patiens with the exacerbation of Chronic Obstructive Pulmonary Disease. J Changchun Coll Tradit Chin. 2006;22: 23.

45 Zhang W, Sun ZJ, Liu JB, Lao WG. Effect of Tanreqing Injection in treatment of 37 paties with the exacerbation of Chronic Obstructive Pulmonary Disease. J New Chin Med. 2006;38: 48-9.

46 Xie YH, Jin FG, Liu TG, Fu EQ, Sun YN. Clinical observation of Tanreqing Injection on acute exacerbation of Chronic Obstructive Pulmonary Disease. J Emerg Syndromes Tradit Chin Med. 2005;14: 291-2.

47 Zhang Y, Li TQ, Wang G, Chang J, Mao B, Wang L, Chi HH, Wan MH. Randomized controlled trial of Tanreqing Injection in treatment of acute exacerbation of Chronic Obstructive Pulmonary Disease (Syndrome of Retention of PhlegmHeat in the Lung). Chin J Evid Base Med. 2004;4: 300-5.

48 Xi R, Zhang ZY, Zhang L, Qu NN, Zhu MD. Study of influence of Xuebijing Injection on CPIS scores and inflammatory markers and immune function of patients with acute exacerbation of Chronic Obstructive Pulmonary Disease. Chin Arch Tradit Chin Med.2017;35: 1240-2.

49 Xu LM. Observation curative effect of Xuebijing Injection in treatment of acute exacerbation of Chronic Obstructive Pulmonary Disease. J Clin Med lit. 2017;4: 2600.

50 Li Y. Clinical observation of Xuebijing Injection on elderly patients with Chronic Obstructive Pulmonary Disease in acute exacerbation. Chin J Exp Tradit Med Form.2016;22: 189-91.

51 Yao LF. Study of influence of Xuebijing Injection in treatment of patients with Chronic Obstructive Pulmonary Disease. J Clin Med lit.2016;3: 4497.

52 Zhu J. Study of influence of Xuebijing Injection on C-Reactive Protein and PCT of patients with acute exacerbation of Chronic Obstructive Pulmonary Disease. Heilongjiang Med J. 2016;29: 864-5.

53 Duan LM, Ning CZ. Observation curative effect of Xuebijing Injection synergistic in treatment of acute exacerbation of Chronic Obstructive Pulmonary Disease. Chin Contin Med Educ. 2015;7: 219-20.

54 Wang XQ. Application of Xuebijing Injection in treatment of acute exacerbation of Chronic Obstructive Pulmonary Disease. Pract Clin J Integr Tradit Chin West Med. 2015;15: 18-9.

55 Zhang H, Gong JH, Zhang JH, Ma JP. Study of influence of Xuebijing Injection on Inflammatory markers and immune function of patients with acute exacerbation of Chronic Obstructive Pulmonary Disease. J Emerg Syndromes Tradit Chin Med. 2015;24: 695-6.

56 Wang N. Influence of Oxidative Stress of Xuebijing Injection on Oxidative Stress of COPD patients and observation on therapeutic effects. West J Tradit Chin Med. 2014;27: 114-5.

57 Mu L. Observation of Xuebijing Injection in treatment of Chronic Obstructive Pulmonary Disease in acute exacerbation. Chin J of Clin Ration Drug Use. 2014;7: 136-7.

58 Fu WH, Zhang TW, Li XB, Zhang S, Zhang YR, Mao LQ, Huang CH. Effect of Xuebijing parenteral solution on the arterial blood gases and lung function of patients with AECOPD. Chin J Gen Pract. 2014;12: 78-9.

59 Shao N. Observation of Xuebijing Injection in treatment of Chronic Obstructive Pulmonary Disease in acute exacerbation. Shandong Med J. 2013;53: 41-2.

60 Zhao L. Observation of Xuebijing Injection in treatment of Chronic Obstructive Pulmonary Disease in acute exacerbation. Tianjin Med J. 2013;25: 46-7.

61 Qiu Q, Pei XJ, Pan Y, Liu Q, Feng Z, Zhang LD, Zhou H. Clinical research of Xuebijing parenteral solution on the coagulation status and lung function of patients with acute exacerbation of Chronic Obstructive Pulmonary Disease. Chin J Crit Care Med. 2013;33: 543-5.

62 Ni XH. Clinical research of Xuebijing in treatment of Chronic Obstructive Pulmonary Disease in acute exacerbation. Med Forum.2012;16: 4537-9.

63 Fan DB, Qin XP, Xu JZ, Bai HH, Zeng YH, Zeng GQ, Yu KS, Yin HY. Clinical observation of Tanreqing Injection in treatment of 86 paties with the AECOPD. Yunnan J Tradit Chin Med Mater Med. 2012;33: 28-9.

64 Li Q, Lian B, Liu WJ. Clinical research of Xuebijing on the lung function and C-reactive protein of patients with AECOPD. Chin J Pract Med. 2012;39: 106-7.

65 Hao TP, Liang FY, Lu YR. Clinical observation of Xuebijing treating acute exacerbation of Chronic Obstructive Pulmonary Diseases. Chin Arch Tradit Chin Med. 2010;28: 2232-3.

66 Tu SY. Clinical curative effect of Xuebijing Injection in curing Chronic Obstructive Pulmonary Disease with acute exacerbation and its effect on endotoxin. Fujian Univ Chin Med. 2010.

67 Lin DB, Yang HL, Chen WT, Li M. Effect of Xuebijing Injection on Chronic Obstructive Pulmonary Disease and blood coagulation function. China Trop Med. 2009;9: 1514-5.

68 Ye L. The clinical significance of Plasma D-dimer, Fibrinogen in patients of AECOPD and Intervention Characteristics and effect evaluation of Xuebijing. Fujian Coll of Chin Med. 2009.

69 Wang B, Li Q, Zhu LQ, Lou JS. Effect of Xuebijing Injection in AECOPD. Tianjin Pharm.2007;19: 43-4.

70 Jia ZR, Yan XL. The value analysis of Danhong Injection treating acute exacerbation of Chronic Obstructive Pulmonary Diseases. Med Forum. 2017;21: 57-8.

71 Ge ZK. The application of Danhong Injection treating acute exacerbation of Chronic Obstructive Pulmonary Diseases. Med Forum.2016;20: 1190-1.

72 Du J. The effect of Danhong Injection in treatment of acute exacerbation of Chronic Obstructive Pulmonary Diseases. China Prac Med. 2014;9: 135-6.

73 Zhang Q. Randomized controlled study in parallel with Chronic Obstructive Pulmonary Disease with acute exacerbation of Danhong Injection combined with western medicine. J Pract Tradit Chin Intern Med. 2014;28: 113-4.

74 Chen Z, Liu Z. Influence of Danghong Injection on TNF-alpha and IL-8 of patients with Chronic Obstructive Pulmonary Disease in acute exacerbation. J Liaoning Med Univ. 2013;34: 23-5.

75 Wang JH, Yan YX, Zhang P. Effect of Danhong Injection in treatment of 62 patiens with the exacerbation of Chronic Obstructive Pulmonary Disease. Hebei J TCM. 2013;35: 1046-7.

76 Zhang L. Clinical effect of Chinese medicine combined with Western medicine in curing Chronic Obstructive Pulmonary Disease with acute exacerbation. Chin Foreign Med Res.2013;11: 26-7.

77 Wang H, Yang LM, Huang L, Chen P. The therapeutic effects of Danhong Injection on the concentration level of CRP, MMP-9 and TIMP-1 in patients with acute exacerbation of Chronic Obstructive Pulmonary Disease. Clin J Chin Med. 2012;4: 51-3.

78 Liu Z, Liu X. Effect of Danhong lnjection on the expression of Interleukin-8 and tumor necrosis factor-α in acute exacerbations of Chronic obstructive pulmonary Disease patients. Chin J Aesthet Med.2010;19: 115-7.

79 Yang RF, Shi XE, Wang CY, Ma XF. Clinical observation of Danhong Injection in treatment of 42 patiens with the elderly exacerbation of Chronic Obstructive Pulmonary Disease. Health Vocat Educ.2010;28: 139.

80 Shi YY. Clinical observation of Danhong Injection treating acute exacerbation of Chronic Obstructive Pulmonary Diseases. J Emerg Syndromes Tradit Chin Med. 2009;18: 1232-3.

81 Tang TZ. Clinical observation on 68 cases of adjuvant therapy with Danhong Injection for Chronic Obstructive Pulmonary Disease. J Pract Tradit Chin Intern Med. 2009;23: 55-6.

82 Chen HC. Influence of Danhong Injection on hemorheology of senile patients with Chronic Obstructive Pulmonary Disease. Henan Tradit Chin Med .2007;27: 26-8.

83 Shen F, Li XJ, Wang P. Clinical efficacy study of using Shenmai Injection as adjuvant therapy for AECOPD patients. Shanxi J Tradit Chin Med. 2017;38: 295-6.

84 Ruan ZW. Clinical Study of Shenmai Injection combined with Salmeterol/Fluticasone in treating AECOPD. J New Chin Med. 2017;49: 40-3.

85 Luo XJ, Ye HB, Zhang TY. Clinical observation of Shenmai Injection combined with Salmeterol/Fluticasone in treating AECOPD. Chin J Rural Med Pharm. 2016;23: 50-1.

86 Xie XX, Li JL, Guo YB. Clinical observation of conventional therapy and Shenmai combined with conventional therapy in treatment of Chronic Obstructive Pulmonary Diseases. Health World. 2016;6: 181-2.

87 Yang JW. Clinical observation of Shenmai Injection in treatment of patients with AECOPD. Summit Forum experience clin emerg. 2015.

88 Huang XL, Zhou YP, Chen DR, Xu GJ. Clinical observation on Shenmai Injection which is one of the compound Chinese traditional medicine preparations in treatment of Chronic Obstructive Pulmonary Disease. Glob Tradit Chin Med. 2014;7: 36-7.

89 Yin ZH, Jia LL. Clinical observation of Shenmai Injection in the treatment of Chronic Obstructive Pulmonary Disease. Med Inf.2013;26: 95-6.

90 Li Y. Clinical observation of Shenmai Injection combined with the third generation cephalosporin antibiotics in treating acute exacerbation of Chronic Obstructive Pulmonary Diseases. Chin J Pharm Econ.2013;8: 100-1.

91 Li C, Li T, Zhao HR. Clinical observation of Shenmai Injection in treatment of elderly Chronic Obstructive Pulmonary Disease. Chin J Misdiagn. 2010;10: 3561.

92 Guo X, Peng DQ. Clinical effect of Chinese medicine combined with Western medicine in curing Chronic Obstructive Pulmonary Disease with acute exacerbation. Chin J Med Drug Appl.2009;3: 153-4.

93 Yang HZ, Dong Y. Clinical observation of Shenmai Injection in treatment of Chronic Obstructive Pulmonary Disease with exacerbation. J Community Med. 2005;3: 76-7.

94 Li JL. Clinical observation of Shenmai Injection in treatment of acute Chronic Obstructive Pulmonary Disease. J Med Res. 2001; 17:37.

95 Ye SH, Zhai YB, Yang JJ. Application significance Reduning Injection in the treatment of acute exacerbation of Chronic Obstructive Pulmonary Disease. J North Pharma.2016;13: 18-9.

96 Dong HZ, Zhang W. The clinical curative effect of Reduning Injection on treating acute exacerbation of Chronic Obstructive Pulmonary Disease Syndrome of Phlegm Heat Obstructing in the lung. J Emerg Syndromes Tradit Chin Med. 2015;24: 1433-5.

97 Tian TL. Clinical study of Reduning treatment of acute exacerbation phase of Chronic Obstructive Pulmonary Disease. J Heze Med Coll .2015;27: 66-8.

98 Zhou JG. Clinical study of Reduning treatment of acute exacerbation of Chronic Obstructive Pulmonary Disease. Pract J Cardiac Cereb Pneum Vasc Dis.2014;22: 83-4.

99 Sun GX, Luo YL, Zhang Q, Zhai JX. Clinical observation of Reduning Injection in treatment of acute exacerbation of Chronic Obstructive Pulmonary Disease. Guide Chin Med. 2012;10: 11-3.

100 Rao ZX. Clinical observation of Reduning Injection in treatment of acute exacerbation of Chronic Obstructive Pulmonary Disease. Zhejiang J Tradit Chin Med. 2012;47: 853.

101 Shen XL, Huang YJ, Xiao QL, Sun HC, Zhang YQ, Zhu QY. Efficacy evaluation of Reduning Injection in treatment of acute exacerbation of Chronic Obstructive Pulmonary Disease (Hot Sputum Pulmonary).Chin J Med Guide. 2012;14: 450-1.

102 Yue YX. Efficacy evaluation of Reduning Injection in treatment of acute exacerbation of Chronic Obstructive Pulmonary Disease. Med Aesthet Beauty. 2014;17: 204.

103 Zhang N, Yu D, Sun JM, Xu Q. Clinical observation of Reduning Injection in treatment of Chronic Obstructive Pulmonary Disease in acute exacerbation of Wind-heat Attacking Lung Type. Med Inf. 2011;24: 293-4.

104 Chen YB. Clinical observation of Reduning Injection in treatment of acute exacerbation of Chronic Obstructive Pulmonary Disease. For all health.2017;11: 144-5.

105 Chen XD, Sun JL, Xie XJ, Wang YS. Clinical study on atorvastatin combined with Ligustrazine Injection in treating exacerbation of Chronic Obstructive Pulmonary Disease. Int Med Hyg Guid. 2014;20: 64-7.

106 Liu Y. Clinical curative effect observation of acute exacerbation with Ligustrazine Hydrochloride Injection in the treatment of Chronic Obstructive Pulmonary Disease. Med Innovation Chin. 2014;11: 17-9.

107 Wang ZF. Effect of TNF-α, IL-8 and CRP of patients with cute exacerbation of COPD treating with Ligustrazine Hydrochloride Injection. Chin J Tradit Med Sci Technol .2013;20: 582-3.

108 Wu BM. Clinical observation of Ligustrazine Injection in adjuvant treatment of AECOPD. Henan Med Res.2013;22: 534-5.

109 Li Q. Clinical observation of Ligustrazine Injection in treatment of acute exacerbation of Chronic Obstructive Pulmonary Disease. Chin J Mod Drug Appl. 2010;4: 152-3.

110 Wu NN. The clinical research of TMP injection to COPD patients in acute aggravating period. Guangxi Univ Chin Med. 2008.

111 Rong JY, Li W. Effect of Ligustrazine Injection on acute exacerbation of Chronic Obstructive Pulmonary Disease. J Hainan Med Univ. 2008;14: 354-6.

112 Effect of blood-gas analysis and plasma fibrinogen of patients with COPD treating with Ligustrazine Injection. J Xiangnan Univ. 2007;9: 24-5.

113 HuangMH, Han Y. Effect of primary outcome indexes of patients with COPD treating with Ligustrazine Injection. Mod J Integr Tradit Chin West Med. 2004;13: 2002-3.

114 Wang HX, Yang ZY, Chen SF. Clinical effect on Chuankezhi Injection combined with salmeterol/ fluticasone in treating exacerbation of Chronic Obstructive Pulmonary Disease. Mod Pract Med. 2017;29: 787-9.

115 Song L, Fan LW. The clinical experience of Chuankezhi Injection in treatment of exacerbation of Chronic Obstructive Pulmonary Disease. Neimonggu J Tradit Chin Med.2012; 28 : 26-27.

116 Liu YK, Fang ZH,Yu ZD, Wang YQ. Clinical observation of Chuankezhi Injection in treatment of 40 patiens with acute exacerbation of Chronic Obstructive Pulmonary Disease. Strait Pharm J.2010;22: 189-90.

117 Cui YD, Wan JG. The improvement of Chuankezhi Injection in treatment of acute exacerbation of Chronic Obstructive Pulmonary Disease. Clin J Tradit Chin Med. 2010;22: 419-20.

118 Liao HL, Sun WG. Clinical observation of Chuankezhi Injection in treatment of acute exacerbation of Chronic Obstructive Pulmonary Disease. Chin J Inf Tradit Chin Med. 2008;15: 77-8.

119 Wang DP, Su HB. Clinical observation of Chuankezhi Injection in adjuvant treatment of acute exacerbation of Chronic Obstructive Pulmonary Disease. Mod Diagn Treat.2014;25: 4236-7.

120 Yuan GH, Sun HC, Wang Y, Jing LJ. Clinical observation of Chuankezhi Injection on acute exacerbation of COPD. Guiding J Tradit Chin Med Pharm.2014;20: 65-7.

121 Zhong RD, Zhou XH, Zhong HP, Zhong CK. Clinical observation of Chinese medicine combined with Western medicine in curing Chronic Obstructive Pulmonary Disease. World Health Dig Med Periodical. 2012;9: 23-4.

122 Qian P. Clinical observation of Chuankezhi Injection in adjuvant treatment of acute exacerbation of Chronic Obstructive Pulmonary Disease. Health Way.2015;14: 109.

123 Han DL, Li FF. Influence of Xiyanping Injections therapy on lung function and inflammation factors of patients with acute exacerbation Chronic Obstructive Pulmonary Disease. Chin J Prim Med Pharm.2012;19: 1078-9.

124 Zhang X, Wang Y, Guo AX. Influence of Xiyanping Injections therapy on inflammation factors and lung function of old patients with acute exacerbation Chronic Obstructive Pulmonary Disease. Clin Med Chin.2014;30: 932-5.

125 Wu DJ. The analysis of curative effect on Xiyanping Injection combined with Piperacillin/ Tazobactam in curing AECOPD. J Mod Med Health.2015;31: 2505-7.

126 Jiang XF. Xi phlogistic flat with antibiotic treatment the clinical effect of acute exacerbation of Chronic Obstructive Pulmonary Disease. Health World. 2016;6: 26-7.

127 Zhang WJ, Chen X. Clinical observation of Xiyanping Injection in adjuvant treatment of elderly acute exacerbation of Chronic Obstructive Pulmonary Disease. Glob Tradit Chin Med. 2011;4: 304-5.

128 Qian YR, Song YQ. The analysis of curative effect of combination therapy of Xiyanping Injection in curing acute exacerbation of Chronic Obstructive Pulmonary Disease. Health World.2013: 3: 63.

129 Zhang YL, Han BY. Effects of Shenfu Injection on the patients with acute exacerbation of Chronic Obstruction Pulmonary Disease. Int J Geriatr. 2017;38: 63.

130 Chi YS. Clinical observation of Shenfu Injection in adjuvant treatment of acute exacerbation of Chronic Obstructive Pulmonary Disease. J Emerg Syndromes Tradit Chin Med. 2015;24: 553-4.

131 Han BY, Li AQ, Tang ZJ, Liu SW, Liu XL, Li X, Qin L. Clinical study on guiding fire to origin method in acute aggravating period of Chronic Obstructive Pulmonary Disease. Mod J Integr Tradit Chin West Med.2013;22: 1825-8.

132 Ren YJ, Li XM, Zhang CM. Clinical observation of Shenfu Injection in the treatment of acute exacerbation Chronic Obstructive Pulmonary Disease. Neimonggu J Tradit Chin Med.2013;29: 58-9.

133. Xu GL. Clinical observation on acute exacerbation of Chronic Obstructive Pulmonary Disease Treating with Shenfu Injection. J Liaoning Univ Tradit Chin Med.2012;14: 193-4.

134 Qin HJ, Liu GP, Zhang F, Shen JM, Sun H. Clinical observation on Shenfu Zhusheye in the treatment of patients with acute exacerbation of Chronic Obstructive Pulmonary Disease. Chin J Clin Med. 2010;17: 659-60.

135 Lian WS, Li WQ, Chen SW, Hu QS. Influence of Shenfu Injection on tumor necrosis factor-α, interleukin- 2 and lung function in patients with Chronic Obstructive Pulmonary Disease at acute exacerbation stage. Chin J Integr Tradit Chin West Med. 2008;15: 149-51.

136 Li ZT, Xin QJ, Zheng SL. Influence of Asarone on Endothelial and Calcitonin Gene-related Peptide in patients with acute exacerbation of Chronic Obstructive Pulmonary Disease. Acta Acad Med Weifang.2017;39: 282-4.

137 Lu N. Clinical observation on 48 cases of Asarone in treatment of Chronic Obstructive Pulmonary Disease at acute exacerbation stage. Chin J Mod Drug Appl. 2015;9: 145-6.

138 Ye Q, Kuang J, Zhu JY. Study of influence of Asarone Injection on blood gas analysis, C-Reactive Protein and PCT of patients with acute exacerbation of Chronic Obstructive Pulmonary Disease. Chin J Lung Dis.2015;8: 64-6.

139 Zhou HY, Wang CH. Clinical observation on Asarone combined with piperacillin-tazobactam in treatment of elderly COPD in acute exacerbation. Chin J Misdiagn. 2009;9: 6824.

140 Dai XZ, Xue W. The clinical effect of a-asarone on treat Chronic Obstructive Pulmonary Diseases with acute exacerbation. Annual Conference of aerospace.2008.

141 Dong GB, Zhang Z, Li CF, Han CY. Clinical observation on 120 cases of Asarone in treatment of Chronic Obstructive Pulmonary Disease. J Binzhou Med Univ. 2008;31: 141-2.

142 Zhang ZH, Wang Y, Zhang F, Cao CM. Clinical observation on 44 cases of α-asarone in treatment of Chronic Obstructive Pulmonary Disease at acute exacerbation stage. Chin J Diffic and Compl Cas.2006;5: 445-6.

143 Xiong SQ, Guo YP, Xiong X. Influence of Astragalus Injection on serum cytokines and lung function in acute exacerbation of Chronic Obstructive Pulmonary Disease. Chin Mod Doct.2013;51: 43-5.

144 Zhang CM. Clinical observation of Astragalus Injection in treatment of Chronic Obstructive Pulmonary Disease at acute exacerbation stage. Yunnan J Tradit Chin Med Mater Med. 2014;35: 54.

145 Tang W. Clinical observation of Chinese medicine combined with Western medicine in curing Chronic Obstructive Pulmonary Disease at acute exacerbation stage. Mod Med J Chin.2012;14: 82-3.

146 Zhang T, Lai CM, Li MQ. Effects of Astragalus Injection on acute exacerbation Chronic Obstructive Pulmonary Disease. Med J West China.2012;24: 33-5.

147 Luo XB, He ZG. Clinical observation of Astragalus Injection in adjuvant treatment of Chronic Obstructive Pulmonary Disease at acute exacerbation stage. Chin J Prim Med Pharm.2008;15: 1213.

148 Zhang C, Liu H, Li HH. Clinical observation on 72 cases of Astragalus Injection in treatment of Chronic Obstructive Pulmonary Disease at acute exacerbation stage. Chin J Tradit Med Sci Technol. 2000;7: 254-5.

149 Zhou Q, Du M, Wang Q. Effects of the Shengmai Injection on clinical symptoms and inflammatory markers of Chronic Obstructive Pulmonary Disease during the acute attack stage. Clin J Chin Med. 2015;7: 12-3.

150 Chen AZ. Clinical observation on 31 cases of Shengmai Injection in adjuvant treatment of COPD at acute exacerbation stage. Chin J Tradit Med Sci Technol. 2013;20: 183-4.

151 Wang Y, Shi CM, Liu Y. Clinical observation of Shengmai Injection in acute exacerbations of Chronic Obstructive Pulmonary Disease. Chin J New Drugs. 2007;16: 1298-300.

152 Gao XL, Cui CB. The effect of Shengmai Injection on systemic inflammatory response syndrome of COPD during the acute attack stage. J Clin Pulm Med. 2006;11: 540.

153 Lv R, Tuo ZN, Cheng YQ, Cai ZH, Ding H. Clinical observation on Shengmai Injection combined with piperacillin-tazobactam in treatment of elderly AECOPD. J Bethune Med Sci. 2015;13: 316-7.

154 Chen HH. 45 cases of Chinese medicine combined with Western medicine in curing COPD at acute exacerbation stage. Zhejiang J Integr Tradit Chin West Med. 2008;18: 171-2.

155 Zhang ZG, Zhang ZH, Zhang LL, Wu YM. Comparative study of clinical effect on AECOPD between Tanreqing Injection and Xiyanping Injection. Pract J Cardiac Cereb Pneum Vasc Dis. 2017;25: 69-71.
