# Supplementary material for: Comparative Efficacy of Chinese Herbal Injections for Treating Acute Exacerbation of Chronic Obstructive Pulmonary Disease: A Bayesian Network Meta-Analysis of Randomized Controlled Trials
Source: Evid Based Complement Alternat Med. 2018 Jul 17;2018:7942936. doi: 10.1155/2018/7942936 (PMC6076913; doi:10.1155/2018/7942936)
Supplement: Supplementary 4 — Table S4: network meta-analysis results of ORs and 95%CIs for rate of clinical effective. [file 7942936.f4.doc]

Table S4. Network meta-analysis results of ORs and 95%CIs for rate of clinical effective

| TRQ+WM |  |  |  |  |  |  |  |  |  |  |  |  |
| --- | --- | --- | --- | --- | --- | --- | --- | --- | --- | --- | --- | --- |
| 0.97 (0.66,1.46) | XBJ+WM |  |  |  |  |  |  |  |  |  |  |  |
| 1.37 (0.87,2.22) | 1.42 (0.85,2.47) | DH+WM |  |  |  |  |  |  |  |  |  |  |
| 1.03 (0.68,1.68) | 1.08 (0.64,1.80) | 0.76 (0.42,1.35) | SM+WM |  |  |  |  |  |  |  |  |  |
| 0.99 (0.60,1.66) | 1.03 (0.58,1.79) | 0.72 (0.38,1.34) | 0.95 (0.51,1.73) | RDN+WM |  |  |  |  |  |  |  |  |
| 1.15 (0.69,1.92) | 1.18 (0.69,2.06) | 0.83 (0.45,1.62) | 1.10 (0.58,2.06) | 1.16 (0.60,2.26) | CXQ+WM |  |  |  |  |  |  |  |
| 0.79 (0.48,1.31) | 0.82 (0.47,1.39) | 0.58 (0.30,1.11) | 0.76 (0.41,1.41) | 0.80 (0.41,1.54) | 0.69 (0.35,1.34) | CKZ+WM |  |  |  |  |  |  |
| 0.85 (0.53,1.39) | 0.88 (0.50,1.59) | 0.62 (0.33,1.15) | 0.82 (0.43,1.45) | 0.86 (0.44,1.67) | 0.74 (0.37,1.42) | 1.08 (0.56,2.02) | XYP+WM |  |  |  |  |  |
| 0.64 (0.36,1.18) | 0.66 (0.36,1.31) | **0.46 (0.24,0.95)** | 0.61 (0.30,1.23) | 0.65 (0.31,1.30) | 0.55 (0.27,1.20) | 0.79 (0.40,1.67) | 0.75 (0.37,1.53) | SF+WM |  |  |  |  |
| 1.13 (0.67,1.97) | 1.16 (0.68,2.11) | 0.82 (0.43,1.63) | 1.09 (0.57,2.02) | 1.14 (0.59,2.24) | 0.98 (0.50,1.98) | 1.42 (0.75,2.83) | 1.32 (0.68,2.66) | 1.76 (0.83,3.69) | XXN+WM |  |  |  |
| 1.27 (0.69,2.47) | 1.32 (0.68,2.59) | 0.92 (0.44,2.04) | 1.23 (0.57,2.54) | 1.29 (0.61,2.73) | 1.11 (0.53,2.34) | 1.61 (0.77,3.20) | 1.50 (0.72,3.16) | 2.00 (0.85,4.47) | 1.13 (0.51,2.42) | HQ+WM |  |  |
| 0.84 (0.40,1.70) | 0.85 (0.40,1.85) | 0.61 (0.27,1.35) | 0.79 (0.35,1.80) | 0.82 (0.37,1.99) | 0.72 (0.31,1.66) | 1.03 (0.46,2.46) | 0.98 (0.41,2.26) | 1.29 (0.52,3.14) | 0.73 (0.31,1.74) | 0.66 (0.26,1.58) | SMI+WM |  |
| **0.24 (0.19,0.30)** | **0.25 (0.18,0.34)** | **0.18 (0.11,0.27)** | **0.23 (0.15,0.34)** | **0.24 (0.15,0.38)** | **0.21 (0.13,0.33)** | **0.31 (0.19,0.48)** | **0.28 (0.18,0.46)** | **0.38 (0.21,0.65)** | **0.22 (0.13,0.34)** | **0.19 (0.10,0.34)** | **0.29 (0.14,0.58)** | WM |

Note: Highlighted results mean there are statistically significant differences between two groups.

ORs, odds ratios; CIs, confidence intervals; TRQ, Tanreqing injection; XBJ, Xuebijing injection; DH, Danhong injection; SM, Shenmai injection; RDN, Reduning injection; CXQ, Chuanxiongqin injection; CKZ, Chuankezhi injection; XYP, Xiyanping injection; SF, Shenfu injection; XXN, Xixinnao injection; HQ, Huangqi injection; SMI, Shengmai injection.
