# Supplementary material for: Comparative Efficacy of Chinese Herbal Injections for Treating Acute Exacerbation of Chronic Obstructive Pulmonary Disease: A Bayesian Network Meta-Analysis of Randomized Controlled Trials
Source: Evid Based Complement Alternat Med. 2018 Jul 17;2018:7942936. doi: 10.1155/2018/7942936 (PMC6076913; doi:10.1155/2018/7942936)
Supplement: Supplementary 5 — Table S5: network meta-analysis results of MDs and 95%CIs for lung functions. [file 7942936.f5.doc]

Table S5. Network meta-analysis results of MDs and 95%CIs for lung functions

| **FEV_1_/FVC** | | | | | | | | | | |
| --- | --- | --- | --- | --- | --- | --- | --- | --- | --- | --- |
| TRQ+WM | -1.9 (-11.72,7.67) | 4.01 (-2.79,11.55) | -1.54 (-10.39,7.24) | -3.07 (-12.89,6.93) | -1.19 (-27.54,26.7) | 1.95 (-15.05,19.16) | 4.52 (-6.2,15.17) | 0.68 (-9.24,10.98) | 1.47 (-15.6,18.06) | **5.18 (0.37,10.24)** |
| -3.15 (-16.96,10.16) | XBJ+WM | 5.99 (-3.82,16.08) | 0.32 (-10.85,11.59) | -1.22 (-13.09,11.15) | 0.76 (-26.61,29.3) | 3.94 (-14.55,22) | 6.47 (-6.36,19.32) | 2.61 (-9.11,14.97) | 3.43 (-14.81,21.62) | 7.12 (-1.09,15.74) |
| -1.85 (-13.96,10.63) | 1.43 (-15.03,18.33) | DH+WM | -5.68 (-14.53,3.27) | -7.13 (-17.17,2.71) | -5.29 (-31.66,22.44) | -2.14 (-19.37,15.11) | 0.48 (-10.68,11.41) | -3.35 (-13.47,7.08) | -2.59 (-19.76,14.13) | 1.15 (-4.19,6.44) |
| -6.34 (-14.76,2.29) | -2.92 (-17.32,11.27) | -4.46 (-17.51,8.01) | SM+WM | -1.35 (-13.1,9.66) | 0.59 (-26.79,28.49) | 3.6 (-14.37,21.4) | 6.22 (-6.42,18.38) | 2.36 (-9.25,14) | 3.24 (-14.95,20.3) | 6.83 (-0.54,14.19) |
| -6.34 (-16.43,3.82) | -2.91 (-17.87,12.01) | -4.39 (-18.42,9.33) | 0.08 (-10.76,10.62) | RDN+WM | 2.26 (-25.24,30.6) | 4.95 (-13.31,23.74) | 7.72 (-5.84,20.47) | 3.72 (-8.52,16.64) | 4.59 (-13.92,22.45) | 8.29 (-0.12,16.98) |
| -1.32 (-28.78,26.92) | 2.14 (-27.79,31.79) | 0.64 (-29.5,29.73) | 5.24 (-22.66,32.75) | 5.04 (-23.45,32.94) | CXQ+WM | 3.17 (-28.09,33.3) | 5.5 (-22.86,33.07) | 1.81 (-27.11,29.02) | 2.43 (-28.21,33.17) | 6.45 (-20.84,32.42) |
| 0.17 (-16.73,16.86) | 3.48 (-17.07,23.65) | 2.14 (-17.77,20.7) | 6.31 (-10.62,23.46) | 6.43 (-12,24.61) | 1.09 (-30.25,33.67) | CKZ+WM | 2.63 (-16.45,21.46) | -1.2 (-19.95,17.33) | -0.66 (-23.14,22.17) | 3.34 (-13.18,19.64) |
| 4.38 (-5.56,14.42) | 7.78 (-7.57,22.49) | 6.25 (-7.64,19.81) | **10.68 (0.26,21.1)** | 10.66 (-1.03,22.34) | 5.68 (-22.84,34.33) | 4.38 (-13.52,21.91) | XYP+WM | -3.81 (-16.88,9.52) | -3.04 (-22.07,15.8) | 0.66 (-8.98,10.62) |
| -0.96 (-12.5,10.52) | 2.31 (-13.92,18.42) | 0.66 (-13.84,15.67) | 5.32 (-6.65,17.39) | 5.28 (-7.84,18.57) | 0.14 (-28.88,29.27) | -1.28 (-19.22,17.6) | -5.37 (-18.63,7.8) | SF+WM | 0.66 (-18,18.61) | 4.54 (-4.57,13.18) |
| -5.01 (-13.9,4.42) | -1.57 (-16.28,12.68) | -3.06 (-16.02,9.71) | 1.37 (-8.05,11.03) | 1.39 (-9.66,12.1) | -3.62 (-31.51,23.74) | -4.91 (-22.43,12.16) | -9.28 (-20.07,1.51) | -3.93 (-15.88,8.47) | HQ+WM | 3.73 (-12.19,20.23) |
| 4.31 (-1.03,9.89) | 7.51 (-4.93,20.2) | 6.18 (-4.86,16.86) | **10.6 (4.1,17.23)** | **10.57 (2.32,18.92)** | 5.48 (-21.82,32.5) | 4.11 (-11.41,19.97) | -0.04 (-8.21,8.19) | 5.35 (-4.73,15.47) | **9.23 (2.06,16.28)** | WM |
| **FEV_1_%** | | | | | | | | | | |

Note: Highlighted results mean there are statistically significant differences between two groups.

MDs, mean differences; CIs, confidence intervals; TRQ, Tanreqing injection; XBJ, Xuebijing injection; DH, Danhong injection; SM, Shenmai injection; RDN, Reduning injection; CXQ, Chuanxiongqin injection; CKZ, Chuankezhi injection; XYP, Xiyanping injection; SF, Shenfu injection; XXN, Xixinnao injection; HQ, Huangqi injection; SMI, Shengmai injection.
