# Supplementary material for: Comparative Efficacy of Chinese Herbal Injections for Treating Acute Exacerbation of Chronic Obstructive Pulmonary Disease: A Bayesian Network Meta-Analysis of Randomized Controlled Trials
Source: Evid Based Complement Alternat Med. 2018 Jul 17;2018:7942936. doi: 10.1155/2018/7942936 (PMC6076913; doi:10.1155/2018/7942936)
Supplement: Supplementary 6 — Table S6: more details about the product information of 12 CHIs. [file 7942936.f6.doc]

based on specification and literature search

|  |  |
| --- | --- |
|  |  |
|  |  |
|  |  |
|  |  |
|  |  |
|  |  |
|  |  |
|  |  |
|  |  |
|  |  |
|  |  |
|  |  |

Table S6. More details about the product information of 12 CHIs

| Injection name | Raw materials | Labeled efficacy | Indications | Adverse drug reactions |
| --- | --- | --- | --- | --- |
| Tanreqing injection | *Scutellariae Radix*, Bear bile powder, Cornu gorais，*Lonicerae Japonicae Flos, Forsythiae Fructus* | clearing heat-toxin, dissipating phlegm | Phlegm heat obstruct lung syndrome, such as fever, cough, expectoration, thirst, redness of tongue, and yellow fur; acute bronchitis, acute pneumonia (early) | Dizziness, nausea, vomit, pruritus, skin rash, fever, chest congestion, edema, phlebitis, anaphylactic shock, dyspnea |
| Xuebijing injection | *Carthami Flos, Paeoniaeradix Rubra, Chuanxiong Rhizoma, Salviae Miltiorrhizae Radix et Rhizoma, Angelicae Sinensis Radix* | Expelling blood stasis, removing toxic substance | Febrile diseases, such as fever, shortness of breath, palpitation, irritability, etc; systemic inflammatory response syndrome induced by infection | Pruritus, chest congestion, palpitation, dyspnea, anaphylactic shock |
| Danhong injection | *Salviae Miltiorrhizae Radix et Rhizoma, Carthami Flos* | Promoting blood circulation for removing obstruction in collaterals | Chest pain and stroke caused by blood stasis obstruction and including chest pain, chest tightness, heart palpitations, facial paralysis, dysphasia, activity inconvenience and other symptoms; coronary heart disease, angina pectoris, myocardial infarction, blood stasis type pulmonary heart disease, ischemic encephalopathy, cerebral thrombosis | Dizziness, headache, palpitation, fever, flushing, skin rash, nausea, diarrhea, anaphylactic shock |
| Shenmai injection | *Radix Ginseng Rubra, Radix Ophiopogonis* | Tonifying Qi and preventing exhaustion, nourishing Yin and generating body fluid, activating pulse | Shock, coronary heart disease, viral myocarditis, chronic pulmonary heart disease and neutropenia with deficiency of Qi and Yin; improve the immune function of patients with tumors, combined with chemotherapy to enhance the curative effect and reduce the toxic and side effects | Flushing, pruritus, dizziness, headache, chest congestion, palpitation, shortness of breath, anaphylactic shock |
| Reduning injection | *Artemisiae Annuae Herba, Lonicerae Japonicae Flos, Gardznize Fructus* | Clearing heat, dispelling wind, removing toxic substance | Cold due to exogenous wind-heat, marked by high fever, headache, body pain, cough, phlegm yellow; upper respiratory tract infection and acute bronchitis | Dizziness, chest congestion, xerostomia, diarrhea, nausea, vomit, pruritus, skin rash, dyspnea |
| Chuanxiongqin injection | Ligustrazine hydrochloride | Inhibiting the aggregation of platelets, dilating blood vessels, improving microcirculation | Occluded cerebrovascular diseases such as cerebral circulation insufficiency, cerebral thrombosis, cerebral embolism and other ischemic vascular diseases such as coronary heart disease, vasculitis, etc. | Skin rash, palpitation, gastrointesstinal adverse reactions, shiver, anaphylactic shock |
| Chuankezhi injection | *Epimedii Folium, Morindae Officinalis Radix* | Warming and recuperating kidney *yang*, calm panting and suppress cough | Asthma, bronchial asthma | Not clear |
| Xiyanping injection | Andrographolide sulfonate | clearing heat-toxin, suppress cough and check dysentery | Bronchitis, tonsillitis, bacillary dysentery | Pruritus, skin rash, palpitation, diarrhea, vomit, diarrhea |
| Shenfu injection | *Radix Ginseng, Radix Aconiti Carmichaeli* | Reviving Yang for resuscitation, tonifying Qi and preventing exhaustion | Desertion syndrome caused by excessive Yang Qi desertion (infectious, hemorrhagic and fluid loss shock); deficiency of Yang or Qi with palpitation, cough, stomachache, diarrhea, rheumatism, etc. | Skin rash, dizziness, headache, vomit, hiccup, fremitus, dyspnea, paropsis, tachycardia, urinary retentron, dysfunction of liver |
| Xixinnao injection | Asarone | calm panting and suppress cough, expelling phlegm, sedation, spasmolysis, anticonvulsant | Pneumonia, bronchial asthma, chronic obstructive pulmonary disease | Palpitation, chest congestion, skin rash, dizziness, nausea, vomit, anaphylactic shock |
| Huangqi injection | *Astragali Radix* | Tonifying Qi and strengthening body resistance, pulseinvigorating and heartnourishing, fortifying spleen and disinhibiting dampness | Insufficiency of the heart-qi and blood stasis syndrome of viral myocarditis, cardiac insufficiency; hepatitis with spleen deficiency and dampness syndrome | Drug fever, drug eruption, swelling and redness at the injection site, laryngeal edema, dyspnea, chest congestion, asthma, hypotension, phlebitis, dysfunction of liver, vomit, diarrhea, headache, anaphylactic shock, rapid atrial fibrillation |
| Shengmai injection | *Ginseng Radix et Rhizoma Rubra, Ophiopogonis Radix, Schisandrae Chinensis Fructus* | benefiting *qi* for nourishing *yin*, restoring the pulse for relieving desertion | Palpitation, shortness of breath, limbs cold, sweating characterized by loss of *qi* and *yin*; Myocardial infarction, cardiogenic shock, septic shock | Allergic eruption, sharp pain in the waist and back, ventosity, chest congestion, palpitation, fever, shiver |
